# Bayesian inverse methods for spatiotemporal characterization of gastric electrical activity from cutaneous multi-electrode recordings

**DOI:** 10.1371/journal.pone.0220315

**Published:** 2019-10-14

**Authors:** Alexis B. Allegra, Armen A. Gharibans, Gabriel E. Schamberg, David C. Kunkel, Todd P. Coleman

**Affiliations:** 1 Department of Electrical and Computer Engineering, University of California San Diego, La Jolla, CA, United States of America; 2 Department of Bioengineering, University of California San Diego, La Jolla, CA, United States of America; 3 Division of Gastroenterology, Department of Medicine, University of California San Diego, La Jolla, CA, United States of America; University of Nevada School of Medicine, UNITED STATES

## Abstract

Gastrointestinal (GI) problems give rise to 10 percent of initial patient visits to their physician. Although blockages and infections are easy to diagnose, more than half of GI disorders involve abnormal functioning of the GI tract, where diagnosis entails subjective symptom-based questionnaires or objective but invasive, intermittent procedures in specialized centers. Although common procedures capture motor aspects of gastric function, which do not correlate with symptoms or treatment response, recent findings with invasive electrical recordings show that spatiotemporal patterns of the gastric slow wave are associated with diagnosis, symptoms, and treatment response. We here consider developing non-invasive approaches to extract this information. Using CT scans from human subjects, we simulate normative and disordered gastric surface electrical activity along with associated abdominal activity. We employ Bayesian inference to solve the ill-posed inverse problem of estimating gastric surface activity from cutaneous recordings. We utilize a prior distribution on the spatiotemporal activity pertaining to sparsity in the number of wavefronts on the stomach surface, and smooth evolution of these wavefronts across time. We implement an efficient procedure to construct the Bayes optimal estimate and demonstrate its superiority compared to other commonly used inverse methods, for both normal and disordered gastric activity. Region-specific wave direction information is calculated and consistent with the simulated normative and disordered cases. We apply these methods to cutaneous multi-electrode recordings of two human subjects with the same clinical description of motor function, but different diagnosis of underlying cause. Our method finds statistically significant wave propagation in all stomach regions for both subjects, anterograde activity throughout for the subject with diabetic gastroparesis, and retrograde activity in some regions for the subject with idiopathic gastroparesis. These findings provide a further step towards towards non-invasive phenotyping of gastric function and indicate the long-term potential for enabling population health opportunities with objective GI assessment.

## Introduction

Gastrointestinal (GI) problems are the second leading cause for missing work or school in the US [1], giving rise to 10% of the reasons a patient visits their physician, and costing $142 billion annually [2]. Symptom management is routinely used by primary care physicians, and patients are referred to GI specialists if symptoms persist, which happens most of the time [2]. While pathologic findings can be detected with a blood test, endoscopy, or imaging, oftentimes symptoms cannot be attributed to a medical condition despite appropriate workup. These disorders fall under the umbrella of functional and motility GI disorders such as Functional dyspepsia and gastroparesis (which affects Parkinson’s and diabetes patients [3, 4]). These disorders make up a majority of patient referrals to GI specialists.

The clinical gold standard for diagnosing motility disorders is gastric emptying, which typically involves imaging after ingestion of a meal containing radioactive tracer. However, gastric emptying does not correlate with symptoms [5] and is not associated with symptom improvement [6]: some drugs improve symptoms but not gastric emptying and vice versa [7–9]. In fact, the NIH Gastroparesis Consortium has recently recommended that improvement in gastric emptying not be considered a requirement for clinical drug trials in gastroparesis [5].

The GI system contains smooth muscle cells (SMCs) that are controlled by rhythmically oscillating slow waves, whose spatiotemporal coordination enables peristalsis to propel food through the GI tract [10, 11]. It has been observed during surgery with invasive high-resolution electrical mapping of the stomach surface that direction and speed patterns of the gastric slow wave co-varies with functional GI disorders, such as gastroparesis, chronic nausea and vomiting, and functional dyspepsia [12, 13]. In a normal functioning stomach, the slow wave is characterized by bands of electric-motor activity which initiate in the pacemaker region (near the fundus) and propagate in equipotential rings in the anterograde direction towards the pylorus [11]. It has also been shown that in patients with GI disorder diagnoses, abnormal initiation can occur, where the bands of activity initiate outside the pacemaker region and bifurcate into a retrograde propagating wave and anterograde propagating wave.

Moreover, recent findings show that features of mucosal multi-electrode recordings predict symptom improvement from gastric stimulation [14–16]. This suggests that new opportunities may emerge to phenotype, localize, and treat such disorders, if such information could be extracted non-invasively.

Conventional electrogastrography (EGG), a noninvasive technique for recording the gastric myoelectric activity using electrodes placed cutaneously on the abdominal surface overlying the stomach [17], is attractive in its non-invasiveness and simplicity in interpretation of a single waveform with spectral analysis. However, conventional EGG solely extracts spectral information [18] and it has been shown that spatial abnormalities can occur at normal frequencies and thus go undetected by conventional EGG [19]. This inability to capture spatial abnormalities that co-vary with functional GI disorders, as well as its inability to correlate with symptoms, might explain why conventional EGG is seldom used clinically [20]. Our recent advances with the high-resolution electrogastrogram (HR-EGG) [21], acquired non-invasively from cutaneous multi-electrode arrays, allows for extraction of abdominal wave propagation parameters at every time point (e.g. presence of a wave, propagation direction and speed). We have recently demonstrated that these features correlate with symptom severity in a population of GI patients spanning a wide range of BMI and ages [22]. This is significant, given the lack of association between symptom severity and gastric emptying. The HR-EGG, however, extracts spatial information relative to the cutaneous surface of the abdomen. From volume conduction, the voltages from cutaneous recordings are the results of an average of complex electrical sources from the gastric surface. Moreover, it was shown [12, 13, 23] that retrograde and anterograde waves from one or more sources can arise at the same time in the distal stomach, suggesting that cutaneous spatial analyses will not be able to resolve them. As such, it has yet to be determined if one can develop a fully non-invasive and simple signal acquisition procedure to reliably extract dynamic spatial patterns of gastric surface electrical activity.

In order to address the problem of non-invasively localizing the site of spatial abnormalities on the gastric surface, we here consider developing inverse methods to infer spatiotemporal electrical patterns on the stomach surface based upon multi-electrode abdominal recordings. Ideally this problem could be solved with a standard linear least squares method if the number electrodes in the observation array exceeds the number of unknowns we aim to infer. For instance, if a linear model relating the unknowns *x* to the observation array *y* is governed by a matrix *A*, then it is well-known that the least squares fit $\hat{x}\left(t\right)$ is given by:

$$\begin{array}{c} \hat{x}\left(t\right)={\left(A^{T}A\right)}^{-1}A^{T}y\left(t\right). \end{array}$$ (1)

However, Eq (1) can only be implemented when *A* is full rank. When it is not, e.g. when the number of unknown variables exceeds the number of observations, the problem is considered ill-posed and there are infinitely many candidate solutions which are equally consistent with the data. One way to address this issue is to use Bayesian inference, in which a prior distribution is specified that enforces a unique solution to a regularized model-fitting problem.

**Fig 1:**
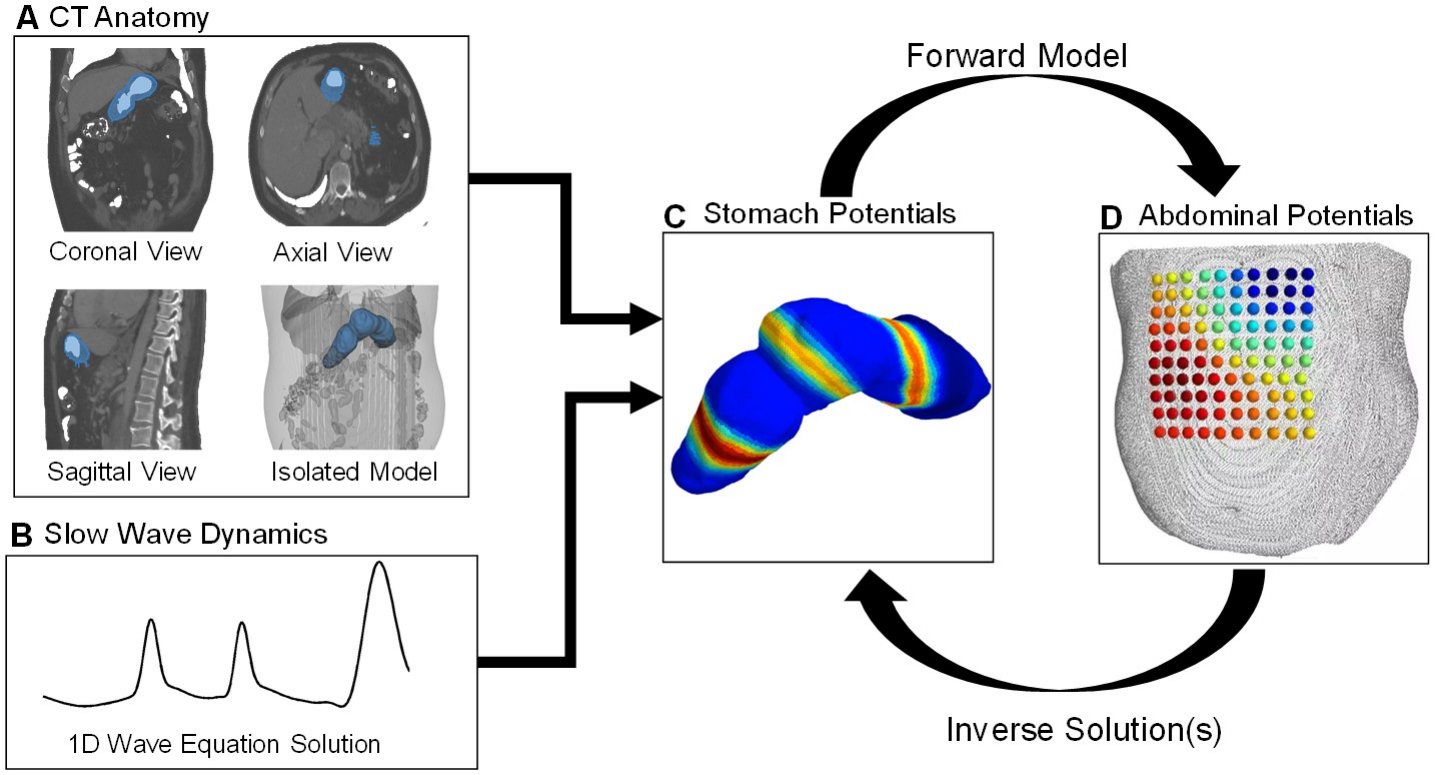
Process workflow. A) Using the 3 standard CT views, a 3D model of the stomach is extracted. B) We develop a spatially inhomogeneous solution to the 1D wave equation, pertaining to propagation down the organoaxial direction of the stomach, with region-specific amplitudes and speeds based upon recent findings in the literature from invasive human recordings. C) The 1D wave equation solution is mapped to the 3D model to generate the dynamic dipole moment solution. D) We solve a forward model to generate the dynamic simulated observations and inject additive measurement noise.

**Fig 2:**
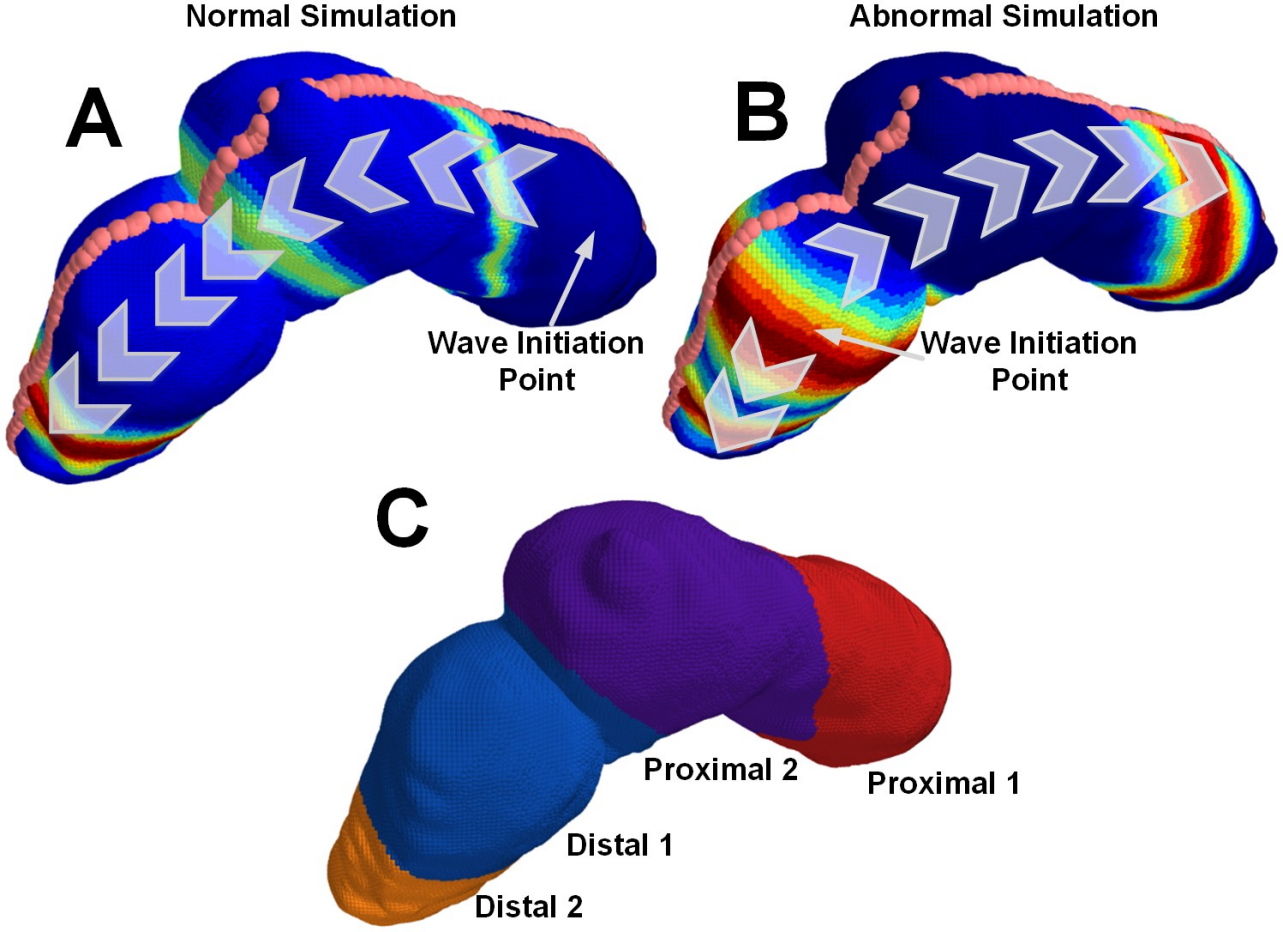
Abnormal and normal simulation propagation directions. A) 3D physiology mapped normal simulation. The wave initiates in the proximal stomach and has only anterograde propagation. B) 3D physiology mapped abnormal simulation. The wave initiates in the distal stomach and has both retrograde and anterograde propagation. C) The different proximal and distal regions of the stomach.

Using computed tomography (CT) scans from human subjects, we simulate gastric surface electrical activity of stomachs for normative (with proximal wave generation and anterograde propagation) and disordered (with distal wave generation and retrograde and anterograde propagation) cases (see Figs 1 and 2). With a forward model to relate the gastric surface potentials to the abdominal surface potentials, we generate a simulated abdominal surface observation array positioned according to the same CT scan. To simplify the computational complexity of the problem, we exploit how circumferential bands of equal potential travel in the organoaxial direction [11] to construct a set of spatial basis functions defined on these annular rings of the gastric surface. These different basis functions represent different numbers of wavefronts per unit space. Thus, estimation of the gastric surface spatiotemporal electric activity is now in terms of estimating the time series of weight vectors.

Even with the simplification of the problem to a set of spatial basis functions, the estimation problem remains ill-posed. As such, we take a Bayesian inference perspective and develop different prior distributions on the time series of weight vectors, each of which pertains to an estimation problem for finding the Bayes optimal point estimate, also termed the Maximum a Posteriori (MAP) estimate. We consider three widely used prior distributions on the time series of weights: Tikhonov regularization, *ℓ*_1_ regularization (which encourages sparsity in the number of active spatial wavefronts and can be solved with the LASSO), and a linear Gaussian state space model (which encourages temporal smoothness and can be solved with the Kalman smoother). In addition, we consider a recently developed [24] group sparsity prior which encourages both sparsity in active spatial wavefronts *and* temporal smoothness. Further, we implement a recently-developed computationally efficient procedure to construct the MAP estimate [25] associated with this group sparsity prior.

We demonstrate that the estimation algorithm pertaining to group sparsity has superior performance in comparison to all other methods, across a range of noise conditions, for both normative and disordered gastric activity. Region-specific wave direction information is calculated and consistent with normal (anterograde propagation in all regions) and abnormal (anterograde propagation on one side of wave origination and retrograde propagation on the other) cases. We apply these methods to cutaneous multi-electrode recordings of two human subjects who have the same clinical description of motor function, but different underlying diagnosed causes (diabetic gastroparesis in subject 1 and idiopathic gastroparesis in subject 2). We find statistically significant wave propagation in all regions for both subjects, anterograde activity in all stomach regions for subject 1, and retrograde activity in some stomach regions for subject 2.

### Previous work

Much of the previous work on solving the inverse problem to infer the gastric slow wave from cutaneous recordings is based on the magnetogastrogram (MGG) [26, 27] which measures the magnetic fields produced by the gastric electric currents. Although the MGG shows promise in its ability distinguish between normative and disordered gastric activity [27], it requires measuring the magnetic field with large environmentally controlled equipment [28, 29]. The HR-EGG signals, in comparison, do not require such shielding and have the potential to be deployed in ambulatory settings. [30].

One previous implementation of an inverse approach to gastric slow wave localization based on simulated abdominal surface electrical measurements utilized a highly detailed but generic ionic channel biophysical model using a generic torso model [31]. This previous approach leveraged classic linear regression (Tihkonov and Tikhonov-greensite) techniques that have been used in the EEG and ECG localization literature [31]. These regression methods attempt to combat the under-determinedness of the problem through the use of regularization either solely in the spatial domain with Tikhonov regression (as in [31, eqn 2]) or through the use of spatiotemporal regularization with Tikhonov-Greensite regularization (as in [31], eqn 3) which uses a spatiotemporal basis [32] to accommodate spatiotemporal continuity in the solution.

## Materials and methods

Key to this study was the development of a three dimensional (3D) time evolving electrical model from which we could generate dynamic simulations. To accomplish this, we first extracted the 3D model of both the stomach and the abdominal surface from a human subject CT. We then developed models of normal and abnormal gastric slow wave electrical activity using a solution to a one-dimensional (1D) wave equation. To map this solution to the 3D stomach model, we sliced it along planes normal to a curve along the stomach which we term the organoaxial curve (see Fig 1). To map the dynamic 3D model of the stomach to the abdominal surface, we solve a standard forward model which assumes that the medium between the stomach surface and abdominal surface is homogeneous with fixed conductivity. The output of this is the simulated abdominal observation array of electric potentials into which we inject additive white Gaussian noise (AWGN) and then apply the aforementioned Bayesian inference techniques to solve the ill-conditioned inverse problem.

### Simulation development

#### 3D physiology

Using 3D Slicer, an open source medical imaging tool [33], we extract an anatomical 3D model of the stomach and abdomen. We placed fiducial points on the abdomen to estimate electrode positions and fiducial lines were drawn on the stomach model surface using a secondary tool (Meshlab) [34]. To simulate the averaging effect of the electrodes, the forward model was solved at several points around the fiducial electrode marker on the abdominal model and averaged together to generate the observation data $Y\in R^{N\times T}$ where *N* is the number of electrodes and *T* is the number of time samples. For this simulation *N* is 100 (a 10x10 grid of electrodes) and *T* is 300 time samples at a sampling rate of 5 samples/second. To map the dynamic simulation of the gastric slow wave to the 3D model of the stomach, three equi-spaced fiducial lines consisting of 120 spatial points were drawn along the surface of the stomach model (see Fig 2 for an example of one of the fiducial lines). From these lines, we derived a series of planes normal to the stomach, and created grouped rings of spatial points. The center-line of the planes forms the organoaxial curve. Onto each one of these rings, we mapped a value of the 1D wave equation solution as a function of time sample. This represents the activation of the normative stomach in equi-potential rings.

#### 1-D wave equation

The gastric slow wave, like the heart, begins with the activation of a group of pacemaker cells on the greater curvature of the stomach [35]. The signal emitted spreads isotropically from the pacemaker region via activation of the interstitial cells of Cajal (ICC), which initiate muscle contractions through the corpus and antrum. Typically, less than 5 simultaneous slow wave wavefronts occur at any time in the human stomach [35].

Due to the continuous nature of the gastric slow wave, and the clear wave-like propagation, we simulated a signal model based on the wave equation. In the normal simulation, wave activity begins in the proximal stomach and the wave direction is entirely anterograde (traveling from the proximal toward the distal stomach). As has been done with other gastric models of the slow wave [21], we ignored circumferential propagation of the serosal slow-wave and solved the following 1D wave equation using a finite difference approach:

$$\begin{array}{c} \frac{\partial^{2}u}{\partial t^{2}}=c{\left(x\right)}^{2}\frac{\partial^{2}u}{\partial x^{2}} \end{array}$$ (2)

where *c*(*x*) is the stomach surface location dependent wave speed, and *u*(*x*, *t*), the 1D wave equation solution, is the amplitude of the wave at each location *x* and time *t*. Gaussian pulses with a width of 35 mm were generated every 20 seconds (0.05 Hz) in the first proximal region of the stomach. The pulse width, in addition to the modulations of its speed and amplitude along the organoaxial direction of the stomach, were chosen to be consistent with the most recent description in the literature for healthy subjects [35–37]. Both the speed and amplitude were highest in the first proximal region (6.0 mm/s, 0.57 mV), followed by a reduction in the second proximal and first distal regions (3.0 mm/s, 0.25 mV), and finally increased in the second distal region (5.9 mm/s, 0.52 mV). See Fig 2 for a description of regions. Mur’s boundary condition was used to ensure the pulses were absorbed into the pylorus rather than being reflected back into the stomach. The Courant-Friedrichs-Lewy condition dictated the temporal step-size to guarantee a converged finite-difference solution.

This same approach is used to develop a dynamic simulation for abnormal initiation. For this part of the simulation, we kept the pulse width as well as speed and amplitude of the wave constant, but modified the initial conditions so that wave patterns were consistent with the findings in invasive recordings [12]: a wave was initiated in the distal stomach and bifurcated into two waves, one propagating anterograde at more distal locations and the other retrograde at more proximal locations. Fig 2 describes the differences between the normal and abnormal initiation simulations.

#### Forward model

**Fig 3:**
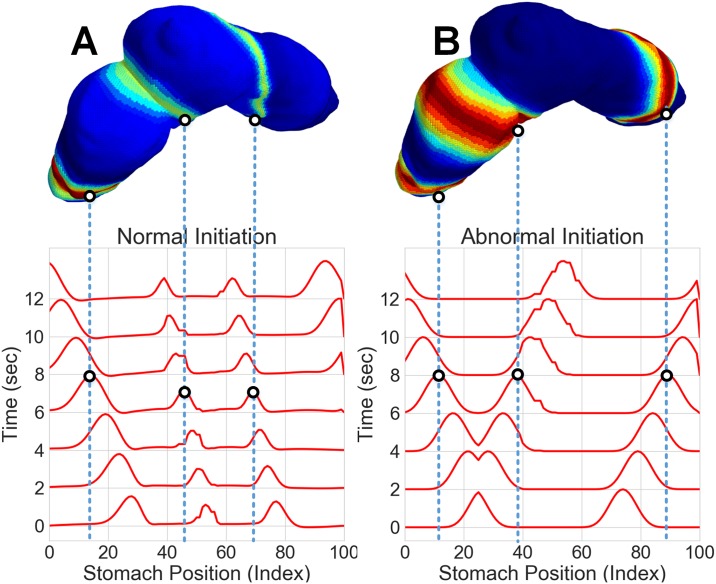
Visualization of source activity mapped to the 3D stomach model. A) 3D stomach model with the normal dynamic simulation mapped at the 6 second time point. The two dimensional time/space plot is shown below the 3D model. B) 3D stomach model with the abnormal dynamic simulation mapped at the 6 second time point. The two dimensional time/space plot is shown below the 3D model.

**Fig 4:**
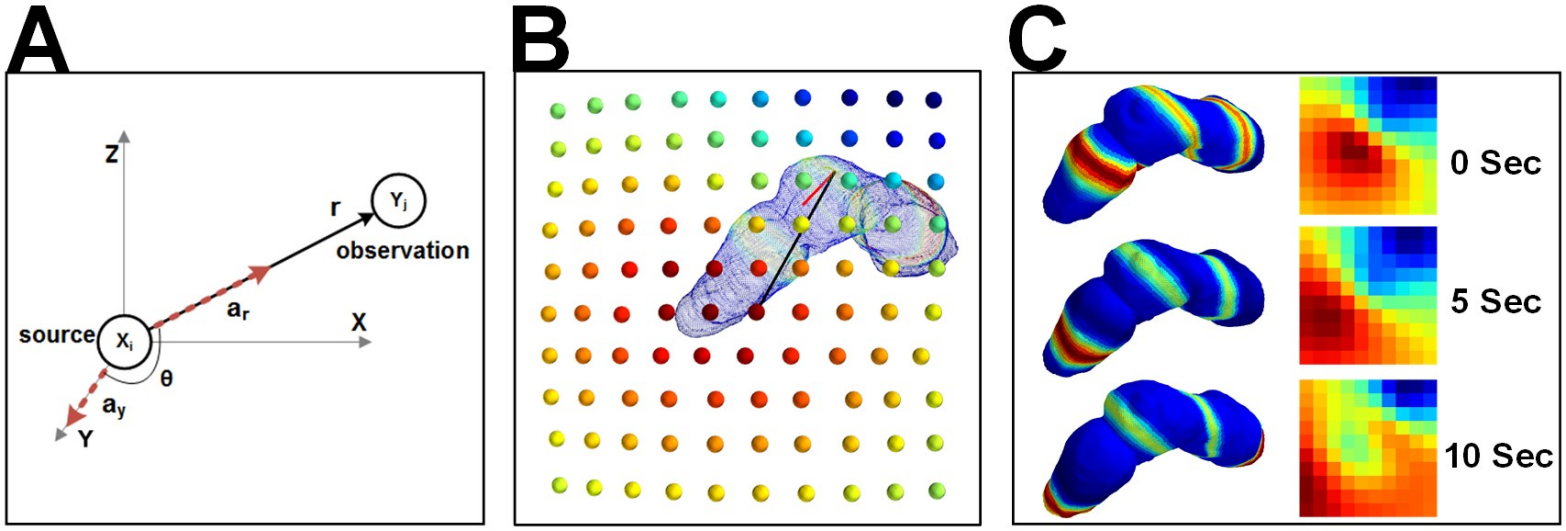
Dynamic dipole moment simulations and forward model. A) Forward model relationship between dynamic dipole moments (X) and observed abdominal surface potentials (Y) B) 3D models of dynamic dipole moments and observed array C) Time evolution of dipole moments and observations for normal simulation.

The 3D stomach model is sliced into annular rings and the 1D wave equation is mapped onto the geometry to represent rings of equipotentials (Fig 3). This solution provides current dipole moments at each point in time, and each dipole is oriented along the organoaxial direction of the stomach [38], as seen in Fig 4A. We compute the estimated EGG signal at each electrode [39] as:

$$\begin{array}{c} y_{n}\left(t\right)=\sum_{i=1}^{D}A_{n,i}x_{i}\left(t\right)+N_{n}\left(t\right) \end{array}$$ (3)

where *y*_*n*_(*t*) is the signal received at each of the *N* electrodes, *A*_*n*,*i*_ is the solution to the forward model at each of the *D* sources on the stomach surface, and *x*_*i*_(*t*) is the dipole moment at each source location and each instant in time. *N*_*n*_(*t*) is additive white Gaussian noise (AWGN) of adjustable variance *σ*^2^, giving rise to adjustable signal-to-noise ratio (SNR) in simulations, in accordance with previous EEG simulation and inverse modeling methods [39].

For the purposes of this analysis, the body is modeled as a homogeneous medium which contains the stomach surface. The solution to the forward model reduces to an attenuation constant which depends on the positions of the source (stomach) and the sensor (abdomen). In matrix notation, the simulated observations are generated as $\vec{y}\left(t\right)=A\vec{x}\left(t\right)+\vec{N}\left(t\right)$, and the solution for the matrix is:

$$\begin{array}{c} A_{n,i}=\frac{\text{cos}\;\theta }{4\pi \sigma r_{n,i}^{2}} \end{array}$$ (4)

where *θ* is the angle between the current dipole (oriented organoaxially) and the observation point on the abdominal surface, *σ* is the tissue conductivity (a constant due to the homogeneous medium), and *r* the straight line distance between the current dipole and the abdominal observation [38].

### Inverse solutions

Although the problem is ill-posed, we aim to take advantage of the fact that the electrical activity of the stomach has key physiologic mechanisms that commonly occur both in normal and diseased states. Specifically, (1) there is a small number of electrically active bands along the surface of the stomach, and further (2) they move continuously over time. We aim to take advantage of this prior physiologic knowledge to utilize Bayesian inference techniques to find an estimate of the gastric electrical activity over time. Since the electrically active bands propagate as waves along the gastric surface, we represent the stomach electrical activity at any point in time as a weighted combination of different spatial basis functions, each of which represents the numbers of wavefronts per unit space. As such, the estimation of electrical activity of the stomach over time, boils down to estimation of the vector of weights over time. We consider different models (prior distributions) of the weights over time which encode different subsets of the two aforementioned physiologic underpinnings of gastric electrical activity. As a baseline, we consider using Tikhonov regularization, the most common form of regularization in inverse problems, which neither encodes physiologic mechanism (1) nor continuous activity over time (2). To solely address (1), the small number of electrically active bands on the stomach surface, we use an L1 regularizer, which promotes sparsity in the number of active weights, and can be solved with the least absolute shrinkage (LASSO) estimation algorithm. To solely address (2), the smooth evolution of the waves, we use a Gaussian state space model, which promotes smoothness overtime and can be solved with the Kalman smoother. To address (1) and (2) in the same estimation method, we consider using a group sparsity model. Further, we use a computationally efficient procedure to solve the MAP estimation problem for this model.

#### Basis functions

For the problem of estimating the stomach surface potentials, we take into consideration how the gastric slow wave contains bands of electrical activity that propagate as rings continuously along the gastric surface [11]. We thus represent the spatiotemporal activity on the gastric surface as a weighted combination of basis functions over space, where the functions are invariant with time but the weights are indexed by time. Under our assumption that the gastric slow wave travels along the organoaxial curve in rings of equi-potential, the bases are solely functions of the position along the one-dimensional organoaxial curve. This curve is visualized in Fig 2 as the points along the top of the geometry.

To construct the set of basis functions, we considered a spatial Fourier basis in which the different sinusoids represent different spatial frequencies, or number of wavefronts that exist per unit space:

$$\begin{array}{c} {\left(H\right)}_{k,i}=\text{cos}\;(\pi i\frac{k}{K}) \end{array}$$ (5)

$$\begin{array}{c} {\left(H\right)}_{k+\frac{K}{2},i}=\text{sin}\;(\pi i\frac{k}{K}) \end{array}$$ (6)

where *i* = 0, …, *D* − 1 represents the spatial index along the organoaxial curve, and *k*/*K* for *k* = 1, …, *K*/2 the relative spatial frequency component.

As such, we solve the problem with respect to basis functions of a line of sources and map the inverse result to the full ring of sources.

$$\begin{array}{c} \vec{x}\left(t\right)=H^{T}\vec{w}\left(t\right) \end{array}$$ (7)

where *H*^*T*^ is the basis matrix (*D* × *K*) and *w*_*k*_(*t*) are the *K* time evolving weights. This reduces the problem to finding an optimum set of weights which are less than the total number of sources. Eq (3) becomes

$$\begin{array}{c} y_{n}\left(t\right)=\sum_{i=1}^{D}A_{n,i}\sum_{k=1}^{K}h_{d,k}w_{k}\left(t\right)+N_{n}\left(t\right) \end{array}$$ (8)

where *h*_*d*,*k*_ is the index into the basis matrix and *w*_*k*_(*t*) is the *k*th weight. Eq (7) simplifies in matrix notation to:

$$\begin{array}{c} \vec{y}\left(t\right)=AH^{T}\vec{w}\left(t\right)+\vec{N}\left(t\right). \end{array}$$ (9)

### MAP estimation for source localization

With the measurement model with respect to the basis representation given by Eq (8), which encodes the statistical model *p*(*Y*|*W*), we formulate the localization problem as one of finding the Bayes optimal point estimate of the time series of weights, which maximizes *p*(*W*|*Y*) over *W* for any set of measurements *Y*. This MAP estimation approach takes into consideration the measurement model *p*(*Y*|*W*) along with the prior distribution *p*(*W*) to identify a solution to our ill-posed problem. The general form of the MAP estimation procedure is as follows:

$$\begin{array}{c} {\hat{W}}_{MAP}=\underset{W\in R^{K\times T}}{\text{arg}\;\text{max}}\;p\left(W|Y\right) \end{array}$$ (10)

where *p*(*W*|*Y*) is the posterior probability of a set of weights *W* given a set of observations *Y*. Using Bayes’ rule

$$\begin{array}{c} p(W|Y)=\frac{p(W)p(Y|W)}{p(Y)}. \end{array}$$ (11)

Since *p*(*Y*) is not a function of *W*, and since the −log function is monotonically decreasing, the maximizer in Eq (10) is equivalent to the minimizer of Eq (12):

$$\begin{array}{c} {\hat{W}}_{MAP}=\underset{W\in R^{K\times T}}{\text{arg}\;\text{min}}\;-\text{log}\;p\left(Y|W\right)-\text{log}\;p\left(W\right). \end{array}$$ (12)

Since the likelihoood *p*(*Y*|*W*) is governed by the forward model and additive Gaussian noise model at the electrodes, from Eq (3), we have that the estimator in Eq (12) becomes:

$$\begin{array}{c} {\hat{W}}_{MAP}=\underset{W\in R^{K\times T}}{\text{arg}\;\text{min}}\;\sum_{t=0}^{T-1}\left|\right|\vec{y}\left(t\right)-AH^{T}\vec{w}\left(t\right){\left|\right|}_{2}^{2}+\lambda \text{pen}\left(W\right) \end{array}$$ (13)

where −log *p*(*W*) is proportional to the penalty term pen(*W*). In each inverse method described, it is this penalty term which will change to support the underlying model assumptions.

Our source estimate for any time *t* is then:

$$\begin{array}{c} \hat{\vec{x}}\left(t\right)=H^{T}{\hat{\vec{w}}}_{MAP}\left(t\right) \end{array}$$ (14)

We evaluate four different priors (pen(*W*)) over the weights, which encode different assumptions about smoothness and sparsity. First, we consider Tikhonov regularization, a classic penalty which was used in previous work [31] and is extensively used in EEG and EKG inverse analysis [40] but does not enforce smoothness or sparseness in the solution. Second, we consider pen(*W*) to be a sum of *ℓ*_1_ penalties over $\vec{w}\left(t\right)$ so that Eq (13) becomes a LASSO problem [41], which encourages sparsity in the solution. Third, we consider pen(*W*) to encode a linear Gaussian state space model so that the solution to Eq (13) becomes a Kalman smoother, which has been applied to the problem of EEG source localization [42] and emphasizes smoothness (i.e. the solution at time *t*_1_ is dependent on the solution at time *t*_0_) in the model. Finally we consider a group sparsity prior, recently developed in [24] for robust spectrotemporal decomposition of time series, for which an efficient and modular solution of Eq (13) with respect to this prior was recently developed in [25]. This group sparsity prior encourages spatial sparsity in the active wavefronts, akin to the LASSO, and encourages temporal smoothness, akin to the Kalman smoother. Each of these methods require finding one or more penalty coefficients λ. Because we generate the ground truth simulation, we find the optimum λ by minimizing the error between the solution found and the simulated data.

#### Tikhonov/Ridge regression

Tikhonov ridge regression makes no assumptions about smoothness or sparsity but is a classic method for solving ill-conditioned problems, by imposing an *ℓ*_2_-norm penalty on *W*:

$$\begin{array}{c} \text{pen}\left(W\right)=\sum_{t=0}^{T-1}||\Gamma \vec{w}\left(t\right){\left|\right|}_{2}^{2} \end{array}$$ (15)

for some square matrix Γ. Unlike the other methods, Tikhonov regression has a closed form solution:

$$\begin{array}{c} {\vec{w}}_{ridge}\left(t\right)={\left({\left(AH\right)}^{T}AH+\lambda \Gamma^{T}\Gamma \right)}^{-1}{\left(AH\right)}^{T}Y\left(t\right) \end{array}$$ (16)

Tikhonov regression has been applied to this problem with some success in previous work on gastric electrical source localization [31, 43], hence it is included here with Γ = *I* for completeness.

#### The LASSO

The LASSO is similar in formulation to Tikhonov regression except that is uses an *ℓ*_1_-norm instead of an *ℓ*_2_-norm. As a result, the LASSO encourages 0-valued weights which enforces sparsity in the solution:

$$\begin{array}{c} \text{pen}\left(W\right)=\sum_{t=0}^{T-1}\left|\right|\vec{w}\left(t\right){\left|\right|}_{1} \end{array}$$ (17)

For this study we use the python Sci-kit Learn implementation of the LASSO [44].

#### The Kalman smoother

The linear Gaussian state space model of the form

$$\begin{array}{c} \vec{w}\left(t+1\right)=\vec{w}\left(t\right)+\vec{m}\left(t\right) \end{array}$$

where each $\vec{m}\left(t\right)$ is a multivariate Gaussian with zero mean and covariance matrix Σ. This gives rise to the Kalman smoother as the MAP solution, which enforces smoothness in time. For Σ = *I*, the penalty is as follows:

$$\begin{array}{c} \text{pen}\left(W\right)=\left|\right|\vec{w}{\left(0\right)||}_{2}^{2}+\sum_{t=1}^{T-1}\left|\right|\vec{w}\left(t\right)-\vec{w}\left(t-1\right){\left|\right|}_{2}^{2} \end{array}$$ (18)

For implementation of the Kalman smoother we used the python pykalman package [45].

#### Group sparsity

We here consider a group sparsity regularization technique that promotes sparsity among groups of coefficients [46] (in our case, weights on the spatial basis functions). For this problem, we establish groups of coefficients over time and impose the penalty on the first differences of the coefficients [47], which imposes temporal smoothness on evolution of the coefficients. As a result, only a sparse subset of the coefficients are non-zero at any given time, and those that are non-zero evolve smoothly over time. Formally, the penalty associated with Eq (13) is given by:

$$\begin{array}{c} \text{pen}\left(W\right)=\sum_{k=0}^{K-1}{\left(\sum_{t=0}^{T-1}d_{k}{\left(t\right)}^{2}\right)}^{\frac{1}{2}} \end{array}$$ (19)

where *d* represents the first differences (in time) of *w*:

$$\begin{array}{cc} & \vec{d}\left(0\right)=\vec{w}\left(0\right) \end{array}$$ (20a)

$$\begin{array}{cc} & \vec{d}\left(t\right)=\vec{w}\left(t\right)-\vec{w}\left(t-1\right),\;t=1,\ldots ,T-1. \end{array}$$ (20b)

The group LASSO can alternatively be viewed as the composition of *ℓ*_1_ and *ℓ*_2_-norms. To see this, first note that:

$$\begin{array}{c} {\left(\sum_{t=0}^{T-1}d_{k}{\left(t\right)}^{2}\right)}^{\frac{1}{2}}=\left|\right|{\vec{d}}_{k}{\left|\right|}_{2} \end{array}$$ (21)

where ${\vec{d}}_{k}≜\left[d_{k}\left(0\right),\ldots ,d_{k}\left(T-1\right)\right]$ is a vector representing the first differences of the coefficients associated with the *k*^th^ basis function. Note the similarity in Eq (20b) combined with Eq (21) to the Kalman smoother penalty in Eq (18), involving an *ℓ*_2_ norm operating on temporal differences of the weight vectors. Using the representation in Eq (21), we can rewrite Eq (19) as:

$$\begin{array}{c} \text{pen}\left(W\right)=\sum_{k=0}^{K-1}\left|\right|{\vec{d}}_{k}{\left|\right|}_{2}=\left|\right|\vec{v}{\left|\right|}_{1} \end{array}$$ (22)

where $\vec{v}≜\left[|\right|{\vec{d}}_{0}{\left|\right|}_{2},\ldots ,||{\vec{d}}_{K-1}{\left|\right|}_{2}]$. Succinctly, the penalty is an *ℓ*_1_-norm of an *ℓ*_2_-norm of time differences.

Under this interpretation, the *ℓ*_1_ norm will allow for only a small number of non-zero elements in $\vec{v}$. Furthermore, *v*_*k*_ = 0 implies that *d*_*k*_(*t*) = 0 for all *t*, which by virtue of Eq (20), further implies that *w*_*k*_(*t*) = 0 for all *t*. Considering only the *k* for which *v*_*k*_ > 0, we can consider the effect of the *ℓ*_2_ norm in Eq (21) for which, in analogy with the Kalman smoother, the non-zero weights ${\vec{w}}_{k}$ will evolve smoothly over time. As a result, we can expect the estimated sources $\hat{X}$ to be composed of a small number of spatial frequency components evolving continuously in time.

We solve the proposed group LASSO problem using a consensus formulation of the alternating directions method of multipliers (ADMM) [48]. A generalized solution framework for using ADMM to estimate latent time-series using sparse regularization is presented in [25].

### Calculation of wave propagation parameters

We extracted estimates of the gastric surface potentials using the MAP estimation procedure in Eq (13) with the group sparsity prior pertaining to Eq (19). With these gastric surface potentials along the organo-axial curve, we identified region-specific wave propagation features of the gastric slow wave. Specifically, we extracted directional information from the phases of the estimated electrical activity on the gastric surface. To determine when the directional information was statistically significant, we utilized a technique called the phase gradient directionality (PGD), which was originally developed in physics and neuroscience communities [49] and was recently employed to describe spatial patterns of GI activity with cutaneous multi-electrode recordings [21].

We extracted wave propagation features of the slow wave from the estimated patterns along the organoaxial curve by first performing the Hilbert transform on each individual estimated source on the curve $\left({\hat{x}}_{i}\left(t\right):i=1,\ldots ,D,t=1,\ldots ,T\right)$ in the array to extract instantaneous amplitude and phase information:

$$\begin{array}{c} {\hat{x}}_{i}\left(t\right)+jHb[{\hat{x}}_{i}\left(t\right)]=a_{i}\left(t\right)e^{j\phi_{i}\left(t\right)},\;i=1,\ldots ,D,\;t=1,\ldots ,T. \end{array}$$ (23)

where *j* is defined to be $\sqrt{-1}$, *Hb* is the Hilbert transform, *ϕ*_*i*_(*t*) is the instantaneous phase of the *i*th source on the organoaxial curve, and *a*_*i*_(*t*) is the instantaneous amplitude of source *i*.

We represented instantaneous phase information as a function of *η* ∈ [0, 1] which parameterizes the organoaxial curve. Discretizing this into *D* points, we have:

$$\begin{array}{c} \phi \left(\eta_{i},t\right)\equiv \phi_{i}\left(t\right),\;i=1,\ldots ,D. \end{array}$$

The spatial gradient of instantaneous phase, ∇_*η*_*ϕ*(*η*, *t*), was constructed at each point *η*_*i*_ along the organoaxial curve. Since the wave velocity vector *v* is normal to contours of constant phase, it satisfies

$$\begin{array}{c} v\left(\eta ,t\right)\propto -\nabla_{\eta }\phi \left(\eta ,t\right). \end{array}$$ (24)

We found the direction of source *i* at position *η*_*i*_ on the organoaxial curve, at time *t*, as

$$\begin{array}{c} \Lambda_{i}\left(t\right)=\text{sign}(-\nabla_{\eta }\phi \left(\eta ,t\right)),\;i=1,\ldots ,D,\;t=1,\ldots ,T. \end{array}$$ (25)

In order to determine if a consistent wave is propagating in a sub-region of the stomach R⊂{1,…,D}, we calculate the PGD in that region, which is the ratio of the norm of the spatially averaged electrode velocities with the spatial average of the norm of electrode velocities:

$$\begin{array}{c} PGD_{R}\left(t\right)=\frac{\|\frac{1}{\left|R\right|}\sum_{i\in R}\nabla_{\eta }\phi \left(\eta_{i},t\right)\|}{\frac{1}{\left|R\right|}\sum_{i\in R}\left\|\nabla_{\eta }\phi \left(\eta_{i},t\right)\right\|},\;t=1,\ldots ,T \end{array}$$ (26)

where |R| indicates the number of elements in the set R and velocities are replaced with −∇*ϕ* by virtue of Eq (24). The PGD is a measure of how aligned the wave velocities at different positions are at any point in time, lies between 0 and 1, and equals 1 for planar waves [49]. Thus one interpretation of the PGD is as a measure of how “close” the activity is to being a plane wave, which is akin to what occurs for a normal slow-wave HR-EGG recordings, exhibiting predominantly anterograde propagation.

In order to control the false discovery rate associated with PGD, we defined statistically significant planar wave propagation to be present when $PGD_{R}\left(t\right)>0.5$ for 1 second or longer (see [21, Fig 2]). A beneficial side effect of computing the PGD is the computation of the wave velocities, from which we can identify anterograde or retrograde propagation at the time points for which the PGD is > 0.5 (i.e. a wave is present). The PGD results for the simulated data are compared in Table 1.

**Table 1:** Percentage of time that PGD > 0.5 for inverse results via simulation type and region.

Data Source	Proximal 1	Proximal 2	Distal 1	Distal 2
*Normal Simulation*	45.7	59.3	82.3	99.0
*Abnormal Simulation*	60.7	80.6	75	72.3
*Noise Alone Simulation*	0.0	0.0	1.67	0.0

Table notes: For the normal simulation, the PGD percentage increases from the proximal to the distal stomach locations. In the abnormal simulation the PGD percentage remains highest in the second proximal and first distal regions, where we detect strong retrograde wave activity. As expected, the noise alone results show no statistically significant wave propagation.

### Human data processing

In simulated data, we can fully characterize the performance of our estimation procedures because the ground truth is known. We here considered applying the group sparsity estimation procedure, e.g. solving Eq (13) with the penalty given by (19), on data collected from two human subjects. Specifically, we collected cutaneous 100-channel EGG recordings on two human subjects for whom CTs were available. Both subjects provided written consent to participate in the study and was part of an ongoing study at the University of California, San Diego, whose institutional review board provided ethical approval (IRB number 141069 “A pilot trial to evaluate the utility of passive, skin-mounted electrodes to monitor the electrical activity of the human digestive system.”). We used a 10 × 10 electrode array with a reference electrode outside of the recording grid. The amplifier was a 256 channel GTec g.HIamp system, sampled at 256 Hz and then down-sampled to 4 Hz. We recorded 90 minutes of EGG data, 30 minutes into which the patient ate a small meal. Prior to analysis, we filtered the data with a band-pass filter with pass band frequencies between 0.015 Hz and 0.25 Hz. We showcase detailed results from one human subject whose CT was used for the aforementioned simulations. We compare summary findings of the human recorded data from both subjects in Table 2. The detailed simulation results and human recording results of the second subject are available in the supporting information.

**Table 2:** Percentage of PGD > 0.5 for inverse results on human recording and region.

Data Source	Proximal 1	Proximal 2	Distal 1	Distal 2
*Subject 1*	17.9	33.7	42.5	97.5
*Subject 2*	24.6	92.1	7.9	95.6

## Results

In both abnormal and normal scenarios we generated a simulated EGG observation array and injected AWGN with varying amounts of noise variance in terms of signal to noise ratio (50 dB SNR down to -4 dB SNR). From these noisy observations, we solved the inverse problem associated with each of the four previously described penalties and evaluated the efficacy of the methods with the correlation coefficient and root-mean-squared error (RMSE) against the true simulated sources. We also provide the 3D results on the geometry as well as a time/space representation of the surface electrical potentials. For the human data, we show the time/space representation of the inverse as well as wave stomach region specific descriptions of propagation pertaining to (a) retrograde vs anterograde propagation and (b) the fraction of time there is a statistically significant wave. These propagation patterns extract phase information across time and utilize the relationship between phase and direction underlying the planar wave equation to extract the PGD measure.

### Simulated data

**Fig 5:**
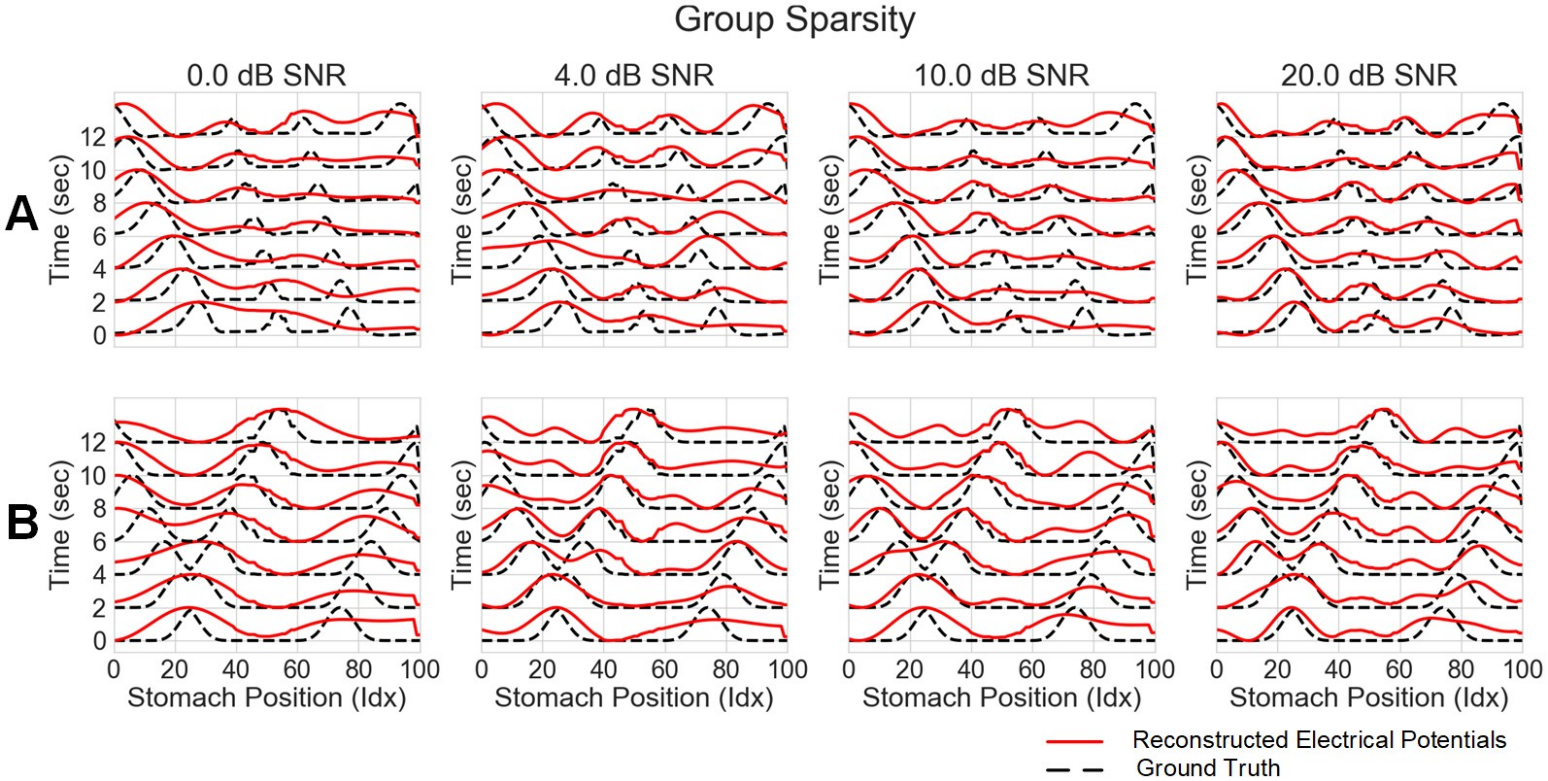
Group sparsity inverse solution on a stomach surface line across noise levels. A) Group sparsity results (electrical potentials) against the ground truth for normal initiation simulation. B) Group sparsity results (electrical potentials) against the ground truth for abnormal initiation. In both normal and abnormal simulations group sparsity is able to reconstruct the ground truth wave pattern even under unrealistically noisy conditions. Additionally the time at which it reconstructs the separate waves in the abnormal simulation is earlier (i.e. the waves are closer together) than in any of the other inverse methods.

Fig 5 shows the results of the group sparsity method against several different noise levels, for both normal (A) and abnormal (B) simulations. The plots for the other methods can be found in the supporting information. In low SNR scenarios, the inverse results visually tracked the ground truth in the distal portions of the stomach (positions 0-50) but struggled to reconstruct the wave in the proximal sections (positions 60-100). However, as the SNR increases, the inverse solution reconstructs the wave in all portions of the stomach. The same findings hold in the abnormal simulation. Additionally, the group sparsity is able to detect the separation of wavefronts in the distal stomach earlier and more clearly than the other methods (see Figs A-C in S1 File). In particular, for the 10 dB noise level, group sparsity is the only method that is able to resolve the two nearby wavefronts in the distal segment at 4 seconds into the recording. Additionally it is able to reconstruct wavefronts occurring in both the proximal and distal segments of the stomach simultaneously.

**Fig 6:**
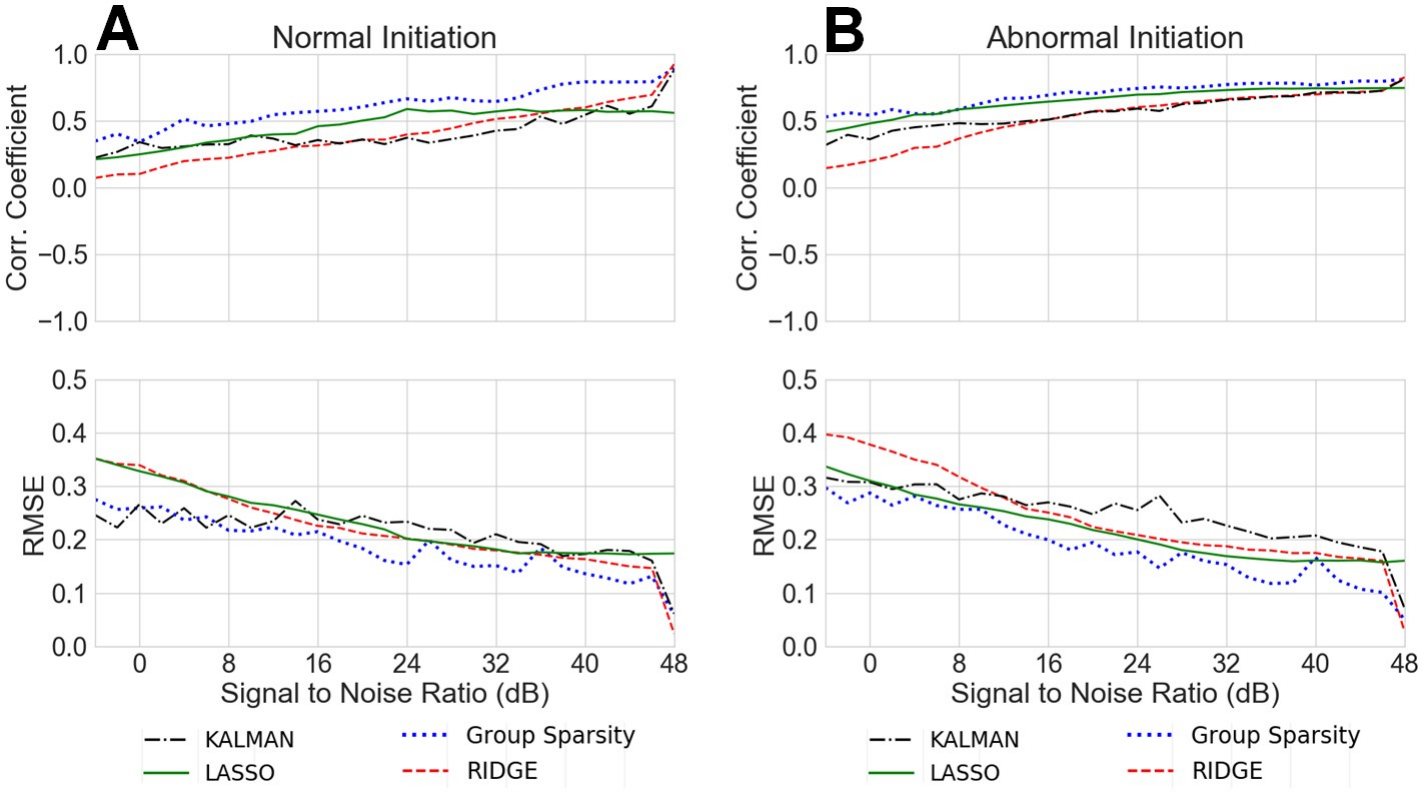
Average correlation coefficient and RMSE for different noise levels (All methods). A) Normal simulation inverse results average correlation coefficient and RMSE as a function of AWGN noise level, B) Abnormal simulation inverse results average correlation coefficient and RMSE as a function of AWGN noise level.

Fig 6 shows the correlation coefficient and RMSE mean across space for all methods, all noise levels, and both normal and abnormal simulations. The group sparsity method remains consistently higher in correlation coefficient and lower in RMSE across all noise levels and in both simulation scenarios. In high additive noise cases (<10 dB SNR), the Kalman smoother based inverse performs similarly to that of group sparsity; however in the abnormal case, LASSO performs slightly better than the Kalman smoother.

**Fig 7:**
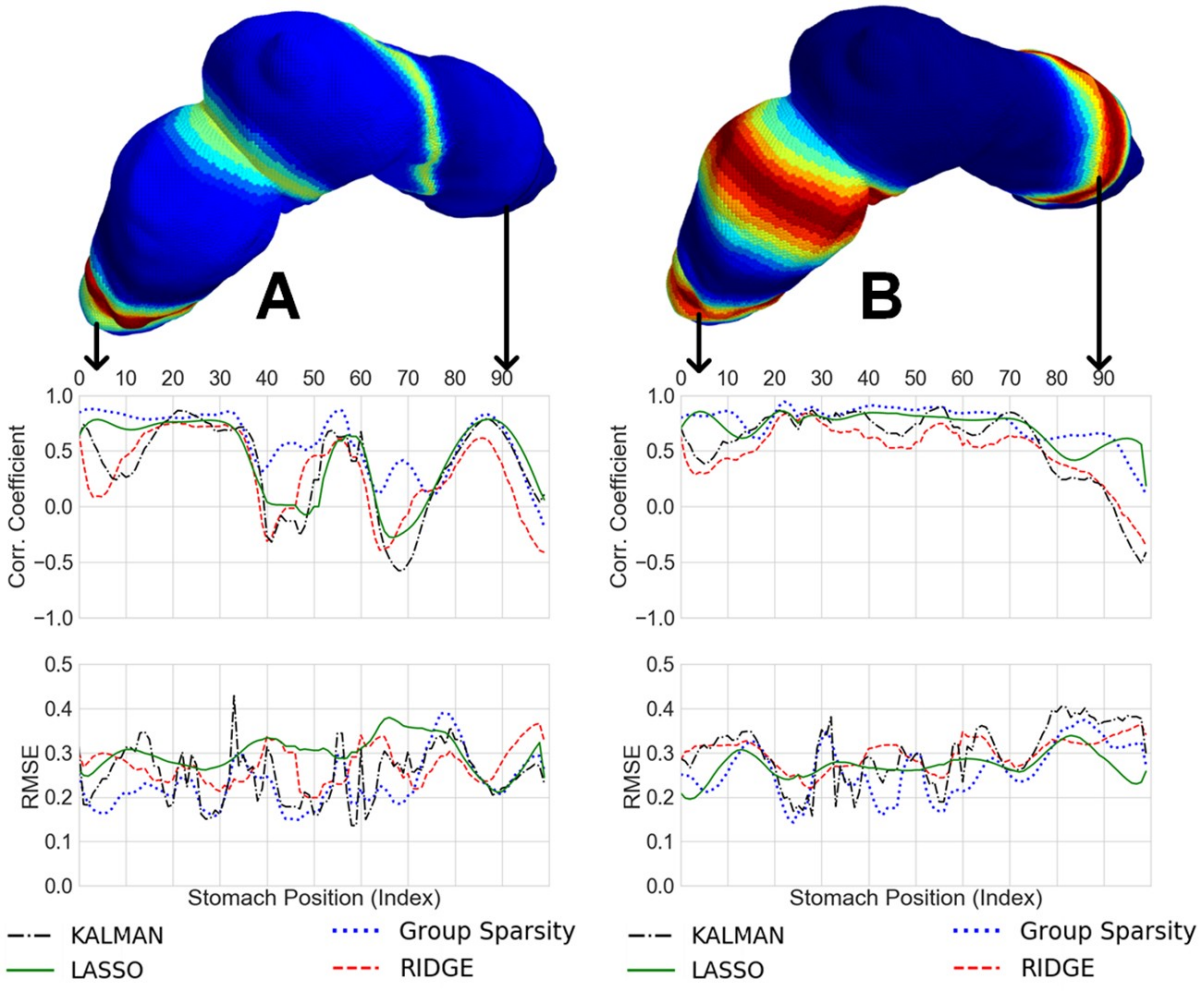
Average correlation coefficient and RMSE for the 10 dB noise level across geometry (All methods). A) Normal simulation inverse results average correlation coefficient and RMSE as a function of geometry, B) Abnormal simulation inverse results average correlation coefficient and RMSE as a function of geometry.

Fig 7 shows the correlation coefficients and RMSEs for all methods at each point along the geometric line in space, for both normal and abnormal simulations, at 10dB SNR. Again, group sparsity has higher correlations and lower errors in both cases. As can be seen in Fig 4, the normal simulation increases in signal amplitude in the distal stomach. The ability to resolve signals in the distal stomach is likely a combination of both the closer proximity to the abdominal surface and the increased signal strength. Specifically, our forward model given by Eq (4) encodes the body conductivity and the distance between source and sensor points and indicates that sources of the stomach with same amplitude that are closer to the abdominal surface will go through less attenuation. The ability to better resolve signals across the entirety of the stomach in the abnormal simulation is most likely due to the constant signal power in the underlying simulation.

**Fig 8:**
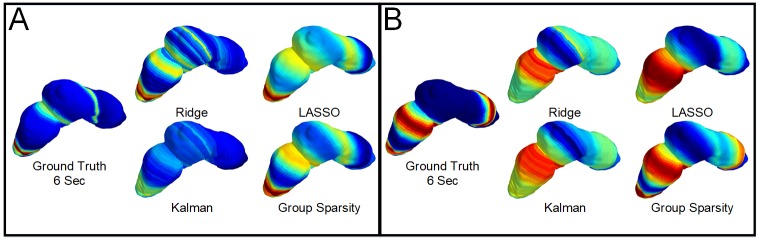
Time snapshot of the inverse results mapped back to geometry. A) Normal simulation at the 10 dB SNR level at the 6 Second time point. B) Abnormal simulation at the 10 dB SNR level and the 6 second time point.

Fig 8 shows the localization results (electrical potentials) mapped back to the geometry for the 10 dB noise level, at 6 seconds into the simulation, in both normal and abnormal cases. The group sparsity method is the only one that resolves the two near wavefronts in the distal stomach, and it is also the only method that can resolve both the activity near the distal stomach and near the proximal stomach simultaneously. The ability of the group sparsity method to resolve near wavefronts and activity across the entirety of the stomach suggests that the joint assumptions of sparsity and smoothness, that it exploits, are critical for reconstruction of the underlying time-evolving gastric electrical activity.

### Region-specific analyses of simulated data

We split the stomach into four sections (proximal 1, proximal 2, distal 1, and distal 2) so that spatial wave propagation parameters can be found in a region-specific manner (see Fig 2C). We analyzed the PGD in each of those regions over time, and found the percentages of time, per region, in which $PGD_{R}\left(t\right)>0.5$. See Table 1.

**Fig 9:**
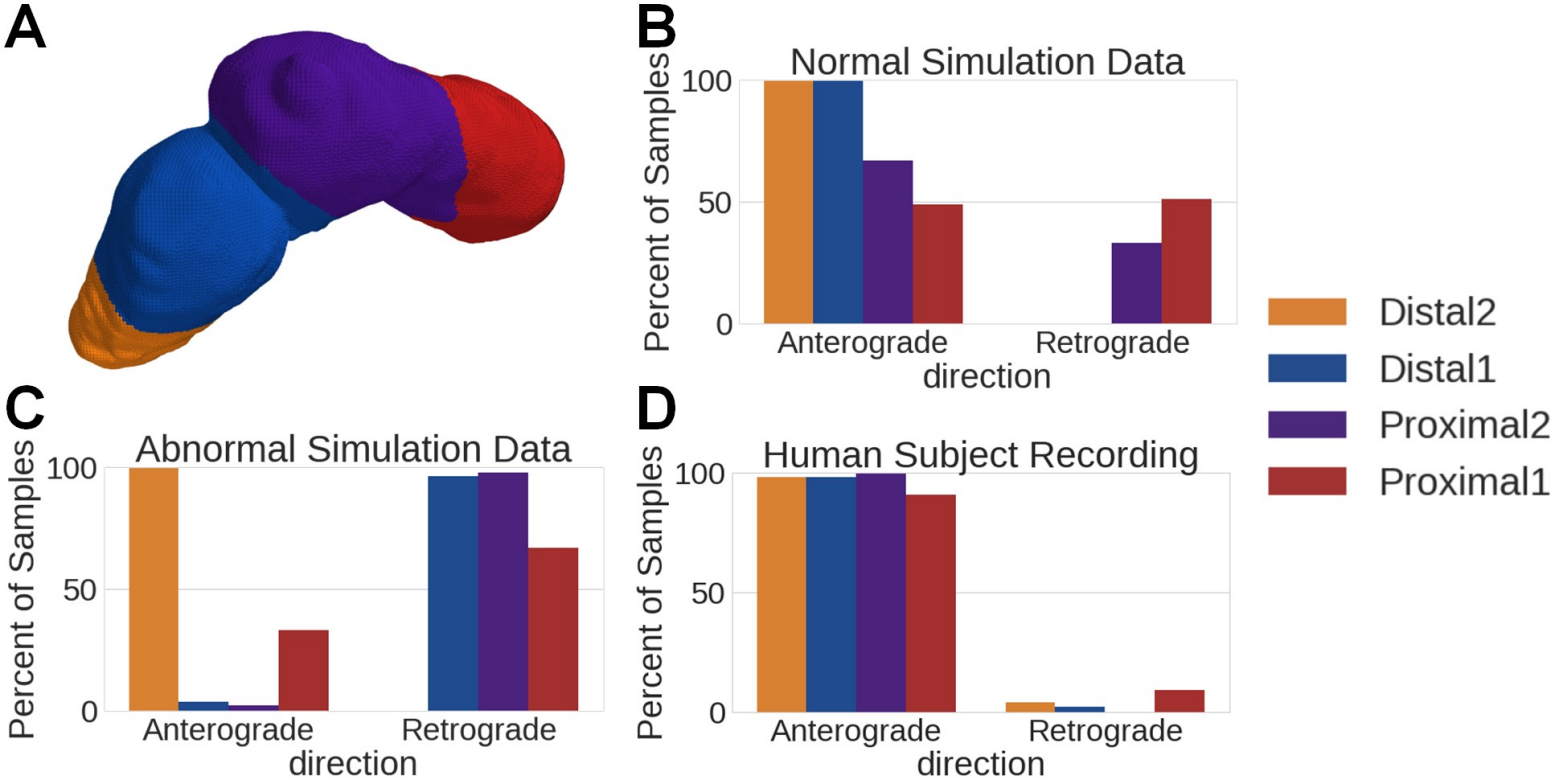
Wave direction histogram by stomach region, for time points in which PGD > 0.5. A) Color coded stomach regions B) Normal data wave directions show strong anterograde wave propagation in all segments of the stomach. C) Abnormal data wave directions show strong retrograde wave propagation in the first distal and all proximal segments, while we see strong anterograde propagation in the distal 2 segment. This aligns with the simulation patterns. D) Human subject data wave directions show anterograde wave movement across all segments of the stomach, the strongest region being the distal 2 segment.

A byproduct of the PGD processing is direction information (anterograde or retrograde) at each point along the organoaxial curve and at each point in time. Fig 9B and 9C showcases region-specific histograms of anterograde vs retrograde propagation whenever $PGD_{R}\left(t\right)>0.5$, for both normal and abnormal simulations. As expected, the normal results have few retrograde waves in each of the four regions, and the abnormal results showcase a large majority of retrograde waves for all regions except distal 2 (where the wave initiation began). In distal 2, a majority of the waves operate in anterograde fashion, as shown by Fig 2. Note that in the abnormal simulation, the signal amplitude remained constant across time, whereas in the normative simulation the signal amplitude varied between the proximal and distal sections of the stomach (see Fig 3). This may explain why the PGD percentages in the proximal sections are higher for the abnormal simulation. More importantly, these results showcase our ability to find the correct direction of propagation in the proximal regions for both retrograde and anterograde, and owing to the large PGD percentages in both contexts, we have confidence that we can determine these directions for a large fraction of the recording.

### Human subject recording

We applied the group sparsity method to a two-minute window of data collected from two human subjects. Both subjects had the same clinical description of motor function, specifically severely delayed gastric emptying in the absence of a mechanical obstruction (severe gastroparesis) with 30% of radiotracer label still in the stomach at 4 hours during a gastric emptying study. Whereas the first subject was diabetic, the most common known cause of gastroparesis due to possible damage of the vagus nerve or enteric nerve cells [50], the second subject was idiopathic, with no known reason for the severe delay in gastric emptying [51].

**Fig 10:**
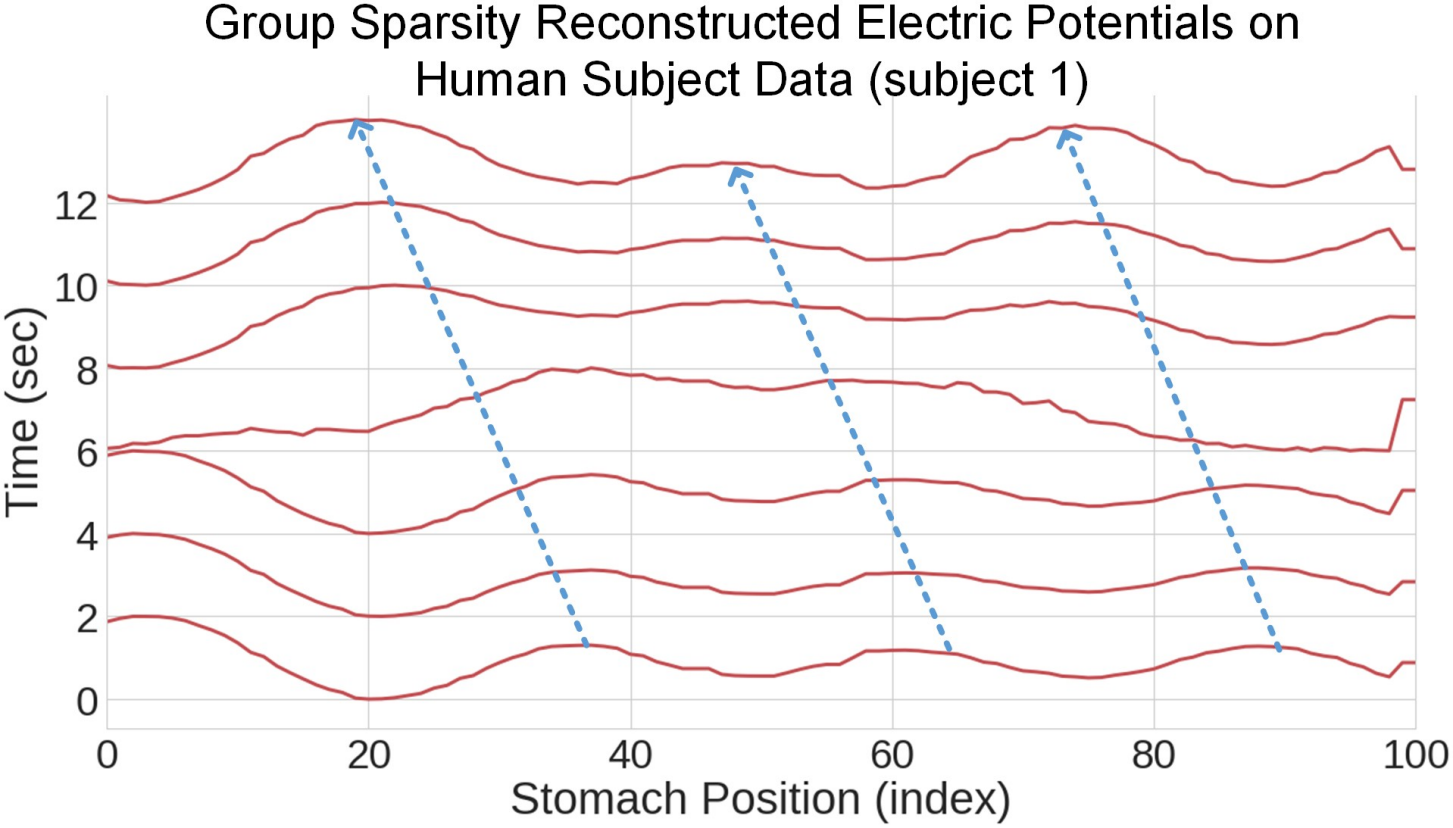
Inverse solution for human subject data. As with the simulated data, this represents twelve seconds of computed inverse data across the sources on the organoaxial curve.

While we do not have ground truth on which to compare the results, the group sparsity method clearly resolves wave activity on the surface of the stomachs, as can be shown in Fig 10 for subject 1. This presence of sustained wave activity is confirmed quantitatively for both subjects in Table 2, which indicates the percentage of time for which a statistically significant wave is present (specifically, $PGD_{R}\left(t\right)>0.5$ for one second).

In human subject 1, all areas of the stomach showed wave propagation, with the distal 2 section showcasing propagation during a significant percentage of the processed data set. The second subject shows strongest activity in the distal 2 segment and the proximal 2 segment. Also of note is that there is very little detected propagation in the distal 1 segment of subject 2, which may be due to the curvature of this particular stomach, as is seen in Fig G in S1 File, versus that of subject 1 Fig 9. Differences in stomach geometry and stomach region proximity to the cutaneous surface are likely explanations for the percentages shown in Table 2. Figs H and I in S1 File show the localization results on the stomach geometry of the two human subjects.

For subject 1, as shown in Fig 9D, almost all waves with PGD >0.5 contain anterograde propagation, strongly suggesting that this control subject does not have abnormal myoelectric function. We also implemented the group sparsity method on white Gaussian noise to verify that our estimation results were not due to chance. As shown in Table 1, the percentage of time for which PGD> 0.5 for the white Gaussian noise simulation is 1.67% in the distal 1 segment and zero everywhere else. See Fig D in S1 File for the reconstructed electrical potentials for the noise alone output.

## Discussion

### Simulation results

Our work provides a novel application of Bayesian methods for spatiotemporal analysis to source localize the gastric slow wave. By exploiting the commonly used assumption of equipotentials along the bands of the organoaxial curve, we were able to parameterize the problem more succinctly (e.g. searching for optimal weights on an over-complete basis). These methods are however generalized, relying only on the relative positions in space of the sources and the sensors, the relationship between the source signal and the sensors (the forward model), and the basis representation. The key assumptions we make with our group sparsity prior are that there are only a few bands of stomach are electrically active at any point in time, and that the electrical activity will evolve smoothly in time. Our method requires knowledge of the stomach and abdominal positioning in 3D space, but otherwise the method is agnostic to specific geometry. To this point, we include an additional human subject for which we developed a model with their CT and applied analogous normal and abnormal simulations for which we evaluated the performance of the group sparsity estimator. For this second subject, we also implemented the group sparsity method on an HR-EGG recorded data segment from the post-prandial segment of data. The results are presented in the supporting information (see Figs E-G in S1 File). As with the first subject, this method is able to reconstruct wave activity in all segments of the gastric surface and resolve the two nearby wave fronts that appear in the distal 2 segment of the gastric surface in the disordered simulation.

Only recently have computationally efficient approaches that combine sparsity constraints and linear state space dynamics been applied to the more richly studied EEG source localization problem [52]. As such, our approach, which combines sparsity with state space modeling, has the potential to be utilized in modern source localization problems for EEG research and beyond.

Previous studies [31] focused primarily on generic physical models of both the torso and the stomach. In this study, the torso and stomach models are taken directly from CT scans of a human subject, and as a result the relationship between the torso (electrode positions) and the stomach in geometry is more akin to a real subject recording. As a result of this, we also see that the methods are better able to reconstruct wavefronts in different regions of the stomach.

Table 1 indicates that for subject 1, the distal segments of the stomach showcase the strongest recovery of wave activity in the normative simulation. Further, Fig 7 showcases that the distal regions have comparatively superior reconstruction performance. This may in part be explained by the fact that for the normative simulation, in accordance with known physiology [35], the signal is stronger in the distal segments of the stomach. This may also be explained by the fact that the anatomical CT for subject 1 shows that the distal segment is closer physically to the abdominal surface. Specifically, by virtue of the denominator of Eq (4), this implies that, absent of considerations of source amplitude, the stomach region closest to the abdominal array (distal 2) more strongly contributes to the HR-EGG.

For the abnormal initiation simulation, we are more able to consistently reconstruct wavefronts across all regions of the stomach, as shown by Table 1. This is likely due to the fact that the wave has consistent signal power, at the same magnitude of the antrum signal power of the normative simulation, across all regions.

For subject 2, the simulated signal activity for the normative simulation still has highest strength signal activity in the antrum. However, the stomach geometry is different from that of subject 1, in particular with more curvature through the proximal and distal segments of the stomach (see Figs H-I in S1 File).

Table A of the supporting information indicates that the proximal 2 segment showed stronger wave activity (75.3%) than the distal 1 segment (64.3%). This suggests that the larger amplitude in the distal 1 region as compared to the proximal 2 region was possibly counterbalanced by the increase in curvature of the stomach, giving rise to variations in the numerator of Eq (4), as compared to subject 1.

We found that across noise levels and simulated conditions in subject 1, the group sparsity approach results in lower squared errors and higher correlation coefficients than the other methods explored. For the abnormal initiation scenario, in low SNR cases the LASSO slightly outperforms the Kalman smoother, and for the normal scenario this is reversed. For abnormal initiation scenario the assumptions about sparsity are perhaps more important than smoothness. However, because the group sparsity approach showed superior performance against all other models (in terms of both mean squared error and correlation coefficient) in all scenarios, it implies that the assumptions of both time smoothness coupled with spatial sparsity are critical. This is further evidenced by the uniqueness of the group sparsity method in separating the close wavefronts during the abnormal initiation, as shown in Figs 5 and 8. It was due to these results that we focused solely on the group sparsity method for subject 2.

These results represent a critical step forward towards objective non-invasive inverse measures for gastric health. While Bayesian inference has been used in EEG and EKG inverse studies, our novel approach aggregates assumptions of spatial sparsity and temporal smoothness into one prior distribution and thus one penalty. Compared to the classic methods, this method shows significant and uniform improvement, thus compelling us to solely use this method when analyzing human subject data.

### Human data and clinical implications

In the human subject recording, we found that after applying our inverse procedure, not only can we identify wave propagation, but we are also able to determine region specific propagation patterns that are consistent with what is known about stomach anatomy and physiology. In the recording for subject 2 who is diagnosed with idiopathic gastroparesis we observe significant and strong retrograde propagation in the proximal regions, as evidenced by 92.1% detected wave activity from Table 2 as well as 100% retrograde activity found in the proximal 2 region (Fig G of S1 File). This differs from subject 1, who had robust wave detection and anterograde activity in all regions (Fig 9). This subject was also involved in a recent clinical study that used the HR-EGG to identify cutaneous spatial patterns [22]. The spatial patterns we found using our inverse method on the gastric surface are consistent with the spatial patterns found on cutaneous HR-EGG analyses from [22]. Specifically, in the inverse method we find anterograde activity in all segments, which aligns with the spatial histogram from cutaneous HR-EGG found in Fig GP-15 in the supplemental materials of [22]. The spatial abnormalities in subject 2 are consistent with findings of loss of ICC cells in idiopathic gastroparesis patients [53], which is known to contribute to gastric myoelectric spatial abnormalities. [12]. That there were more detected spatial abnormalities in an idiopathic gastroparesis subject as compared to a diabetic gastroparesis subject can perhaps be explained by findings of more severe ultra-structural changes in ICC cells and nerves idiopathic gastroparesis patients as compared to patients with diabetic gastroparesis [54].

Understanding the relationship between myoelectric activity in different parts of the stomach can allow for sub-typing of gastric disorders. For instance, antrum and pylorus coordination or lack thereof can help predict (and thus explain) gastric emptying of meals in humans [55]. As such, being able to non-invasively extract myoelectric patterns in stomach sub-types, and evaluate their coordination, can give rise to etiologies of certain GI disorders and suggest therapies. It was recently shown that spatial features from the cutaneous HR-EGG correlate with symptom severity [22]. As such, our approach to identify spatial slow wave abnormalities (such as abnormal initiations discussed here), in region-specific manners, may advance the potential to enable guided therapies, such as ablation [56] or gastric pacing [57, 58] to normalize the slow wave and ameliorate symptoms.

One existing therapy, high frequency gastric electrical stimulation, has been shown to improve symptoms by affecting central control of nausea and vomiting [59]. Moreover, features from invasive electrical recordings on the stomach surface predict which patients respond well to this therapy [15]. This suggests that if this information could be extracted non-invasively, new opportunities exist to phenotype such disorders and assess their response to interventions. In addition, recent efforts to directly modulate gastric electrical activity with an artificial pacemaker have the potential to improve gastric function [60, 61]. However, determining the stimulation location and parameters and confirming the restoration of normal electrical activity requires invasive measurements [62]. Our noninvasive approach could guide these types of targeted therapies for gastric disorders, as has been done successfully in cardiology with identifying and treating arrhythmias [63, 64].

### Limitations and future research

The gastric slow wave is normally active at all times, but triggers more contractions when co-regulatory factors (such as stretch from food ingestion) are present. This initial work used a straight forward dynamic model which did not account for volume changes and region-specific deformations due to food or deformation due to contractions [65, 66]. Further, the increase in contractions due to eating may give rise to stronger electrophysiologic potentials, which was not modeled here. These aspects could be incorporated into both the forward model and the prior for future studies.

There are two further approaches to extend this model. For one, our forward model assumes a homogeneous space with the same conductivity to relate the gastric surface potentials to the recorded abdominal potentials. In reality, there are tissues with different conductivities (e.g. fat and muscle) between the stomach and abdominal surface. Using models that capture this information can be done in future work. Further, our model uses simple one-dimensional wave equation propagation models to represent the spatiotemporal relationship of the gastric electrical activity. Future work can take into consideration cellular compartment models with differential equations, as has been done in other EGG modeling works [67–69]. Validation of these methods using abdominal recordings from a larger group of asymptomatic human subjects as well as those with diagnosed disorders may further give credence to this approach. Lastly, selecting regularization coefficients in a data-dependent manner when using model-fitting procedures that leverage sparsity is a subject for future work [70, 71].

## Conclusion

In this paper, we used CT images from human subjects to develop a basic dynamic dipole model of the electrical activity on the surface of the stomach (the gastric slow wave). We solved a forward model, then corrupted with additive Gaussian noise, to simulate cutaneous multi-electrode recordings. Using these simulated observations, we found the inverse solution by formulating the inverse problem as a MAP estimation problem to find a set of optimal weights on a set of spatial Fourier basis functions. Each method we explored leveraged different assumptions about smoothness (Kalman smoother), sparsity (LASSO), a combination of the two (group sparsity), and no assumptions at all (Tikhonov regularization). We found that in low to no noise environments, Tikhonov regularization is sufficient. However, in noisier environments, the assumptions around smoothness have the most impact and result in lower RMSE and higher correlation coefficients. Additionally, by incorporating a prior distribution for which the MAP estimate takes advantage of both smoothness and sparsity (the group sparsity approach), the results are even further improved. These results uniformly attained the highest performance, for both abnormal simulations as well as normal simulations.

Finally, we applied this approach to an asymptomatic human subject who had previously been imaged with CT. We found statistically significant results for wave activity, as well as region specific wave direction information that is consistent with current knowledge regarding normal gastric myoelectric function [11]. The presented methods may benefit from further studies and validation on mammalian subjects against invasive “gold-standard” methods. While further research is needed to verify this approach on more human subject data against a secondary measure of electrical activity on the stomach surface, these initial results show significant promise towards utilizing a non-invasive technique to localize electrical activity in different regions of the stomach, possibly even in ambulatory settings [30].

## Supporting information

S1 File The results of the other inverse methods (ridge regression, LASSO, and the Kalman smoother) as well as the results from the second human subject are presented here. (PDF)

S2 File The model data and results for subject 1 are included in this zip file. The files contained are described in the README.txt file of the unzipped folder.
(ZIP)

S3 File The results for subject 1 simulations are included in this zip file. The file is described in the README.txt file of S2 File.
(ZIP)

S4 File The model data and results for subject 2 are included in this zip file. The files contained are described in the README.txt file of the unzipped folder.
(ZIP)
