# Supplementary material for: Bayesian inverse methods for spatiotemporal characterization of gastric electrical activity from cutaneous multi-electrode recordings
Source: PLoS One. 2019 Oct 14;14(10):e0220315. doi: 10.1371/journal.pone.0220315 (PMC6791545; doi:10.1371/journal.pone.0220315)
Supplement: S1 File — (PDF) [file pone.0220315.s001.pdf]

Other Inverse Methods

Below are the time/space plots of the reconstructed electrical potentials on the surface of the stomach for ridge regression, LASSO, and the Kalman smoother.

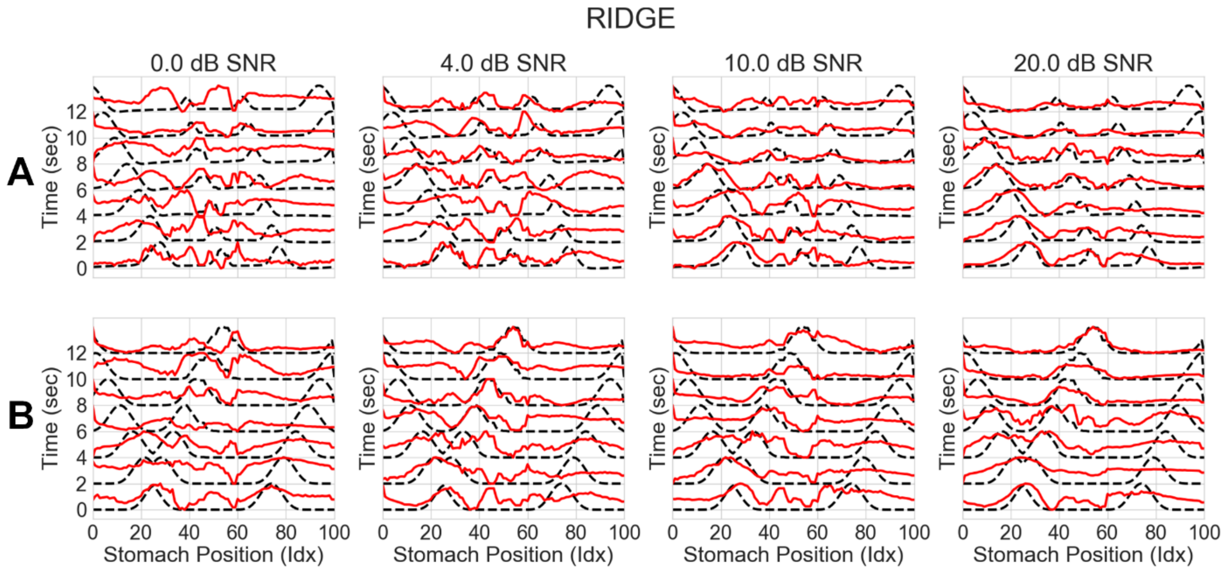

Fig A. Tikhonov Ridge Regression Inverse Solution on a Stomach Surface Line Across Noise Levels A) Ridge regression results (electrical potentials) against the ground truth for normal initiation simulation. B) Ridge regression results (electrical potentials) against the ground truth for abnormal initiation. In both normal and abnormal simulations, ridge regression is not able to fully reconstruct the ground truth wave pattern even under unrealistically strong signal conditions.

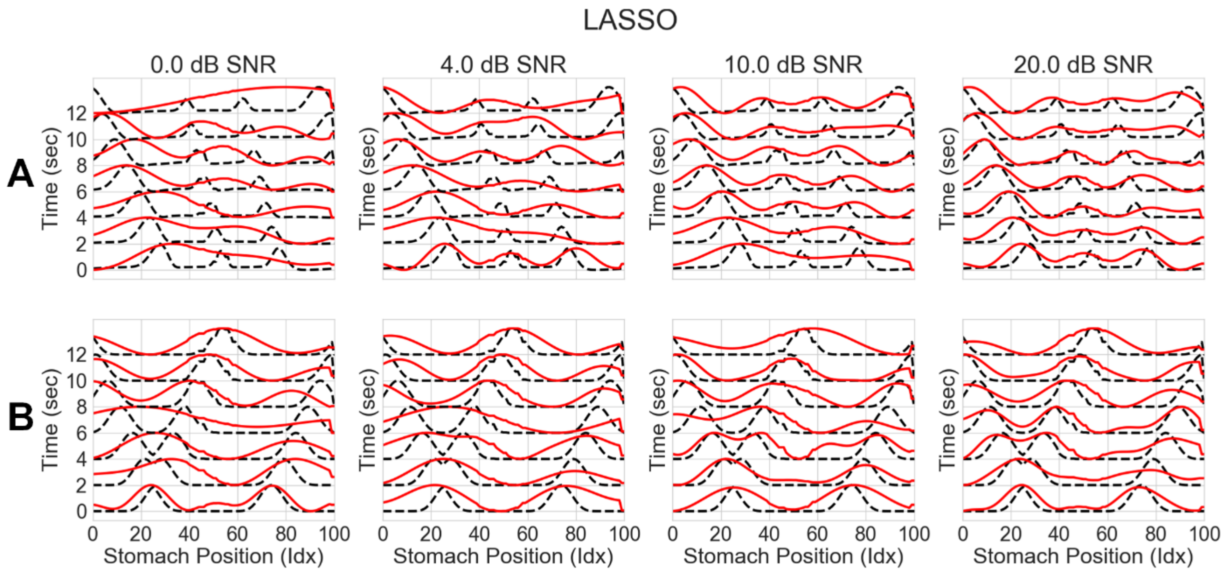

Fig B. LASSO Inverse Solution on a Stomach Surface Line Across Noise Levels A) LASSO results (electrical potentials) against the ground truth for normal initiation simulation. B) LASSO results (electrical

potentials) against the ground truth for abnormal initiation. In both normal and abnormal simulations LASSO is only able to reconstruct the ground truth wave pattern where the signal is the strongest in the distal portions of the stomach. Even for these, it does not perform well in the presence of noise.

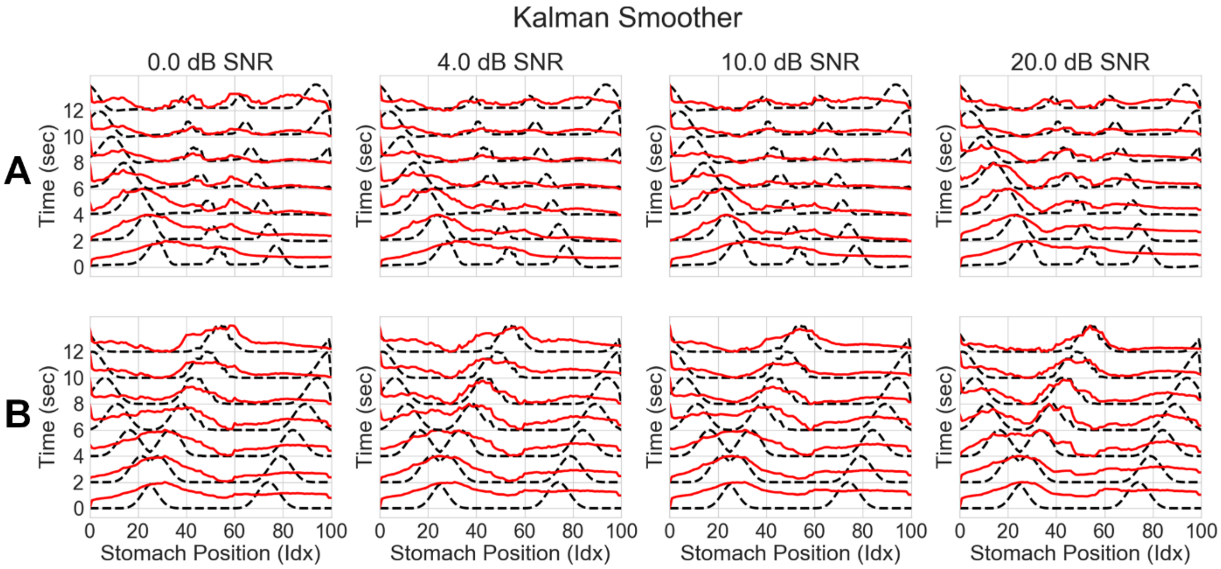

Fig C. Kalman Smoother Inverse Solution on a Stomach Surface Line Across Noise Levels A) Kalman smoother results (electrical potentials) against the ground truth for normal initiation simulation. B) Kalman smoother results (electrical potentials) against the ground truth for abnormal initiation. In both normal and abnormal simulations, the Kalman smoother tracks the ground truth simulation fairly well after the first time point

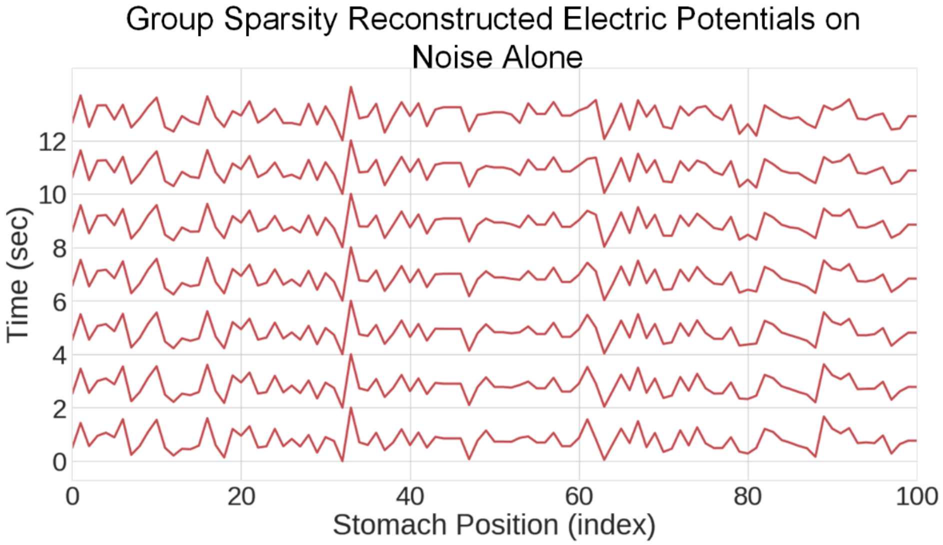

Fig D. Group Sparsity Inverse Solution on the Stomach Surface due to Noise Only The group sparsity inverse reconstructed potentials due to a noise only simulation; no wave activity.

Second Human Subject Results

For basic validation of these methods, we include results from a second human subject who had the same clinical description of motor function (GES at 30% at 4 hours) as the first human subject reported upon in the main manuscript. Below we report the reconstructed potentials for both the simulated waves (Fig E) as well as the actual recording (Fig F), and the histograms of wave direction (anterograde or retrograde) as a function of time and stomach region (Fig G).

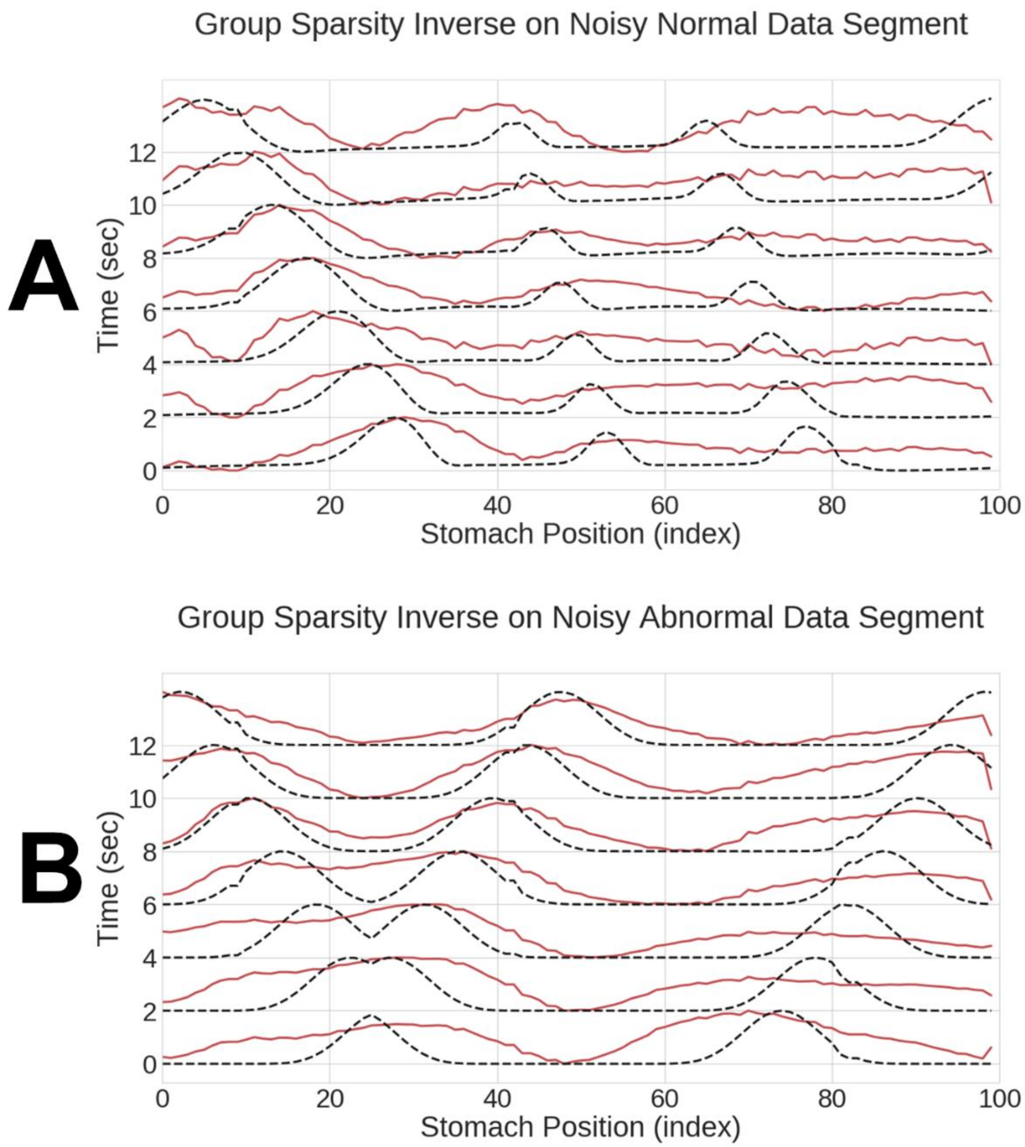

Fig E. Subject 2 Simulation Reconstructed Potentials The time and space reconstructed electrical potentials from the group sparsity inverse for the second subject. (A) is the reconstructed potentials for the normal simulation. (B) is the reconstructed potentials for the abnormal initiation simulation.

Along with the first subject reported in the main manuscript, this subject also was also involved in a recent clinical study that used the HR-EGG to identify cutaneous spatial patterns [1]. Commensurate with what occurred with subject 1, the spatial patterns we found using our inverse method on the cutaneous surface are consistent with the spatial patterns found on

cutaneous HR-EGG analyses from [1]. Specifically, in the inverse method we find strong retrograde activity in both proximal segments, which aligns with the spatial histogram from cutaneous HR-EGG found in Fig GP-8 in the supplemental materials of [1].

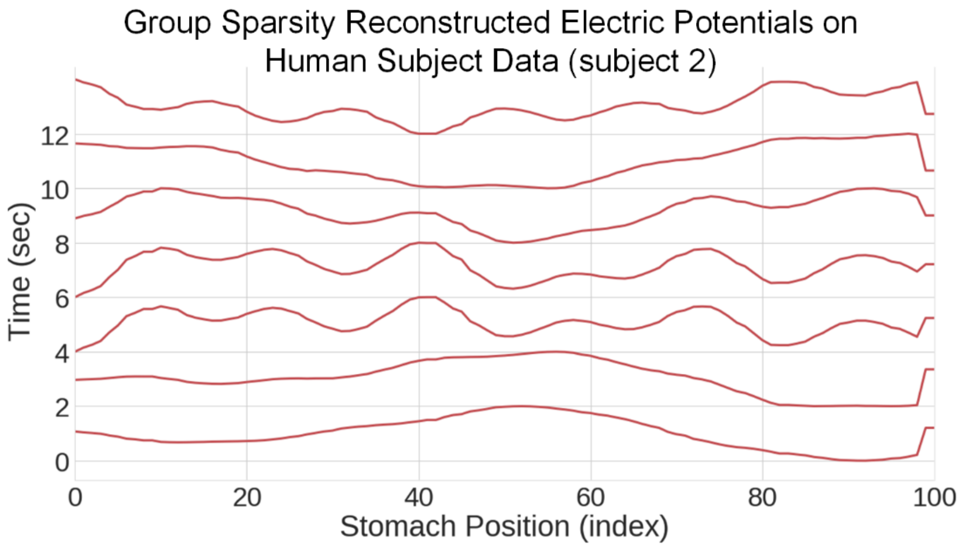

Fig F. Subject 2 Recording Reconstructed Potentials The time and space reconstructed electrical potentials from the second subject recording using the group sparsity inverse

Table A. Percentage of Time that PGD > 0.5 for Inverse Results Via Simulation Type and Region (Subject 2)

**Data Source**	**Proximal 1**	**Proximal 2**	**Distal 1**	**Distal 2**
**Normal Simulation**	48.3	75.3	64.3	84.7
**Abnormal Simulation**	52.7	97.3	95.3	92.3

In human subject 2, all areas of the stomach showed wave propagation, with the second distal section show-casing the highest percentage 84.7% of wave propagation in the normative data set. Strong activity is also present in the proximal 2 segment 75.3%.

Also, of note is that the distal 1 segment shows less activity than that of the proximal 2 segment for this subject. This may be due to the curvature of this particular stomach, as is seen in Fig G, versus that of subject 1 (Fig 9).

Differences in stomach geometry and stomach region proximity to the cutaneous surface are likely explanations for the percentages shown in Table 2.

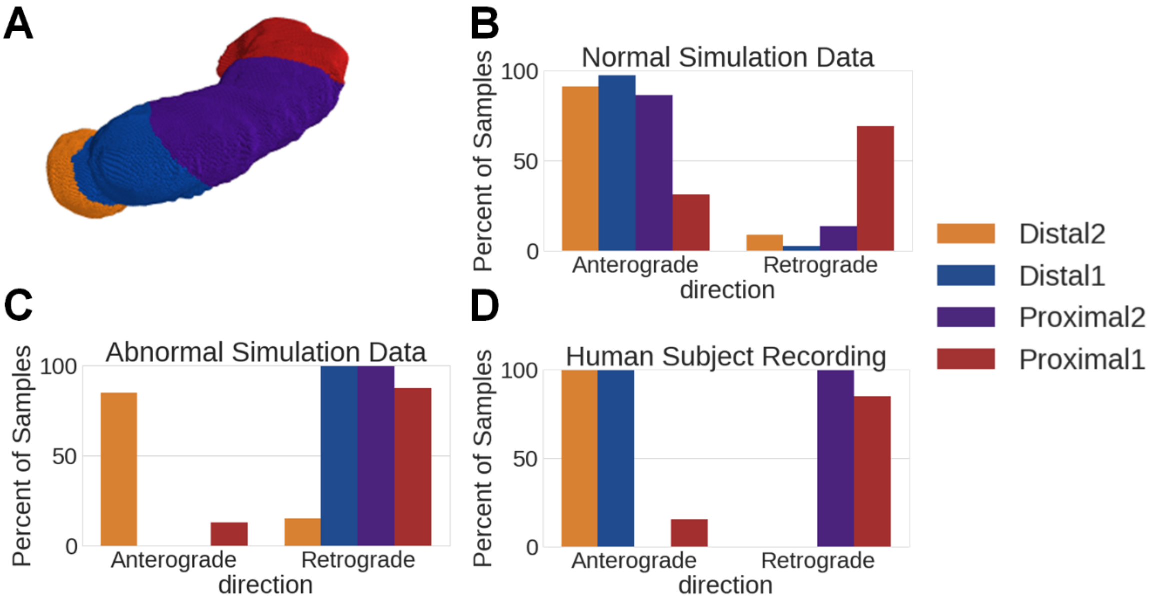

Fig G. Subject 2 Direction Histograms (A) the second subject stomach geometry mapped into 4 regions. (B) the direction histogram indicating entirely anterograde direction for the normal simulation. (C) the direction histogram for the abnormal simulation. (D) the direction histogram for the recorded subject data

In terms of geometry, each stomach and abdominal CT represent different geometric relationships between the abdominal surface and the gastric surface. The level of curvature in different stomach regions, and the effect that has on the propagation of electrical activity to the electrodes on the abdominal surface, can play a role. Further, eq 4 pertaining to the forward model contains a cos θ in the numerator, where θ is the angle between the current dipole (oriented organoaxially) and the observation point on the abdominal surface. As such, if we have strong curvature, cos θ can go through significant changes throughout that region and so the superposition principle can result in the cutaneous electrode array containing interference between nearby dipole sources. Altogether, this would result in a reduction in signal to noise ratio and thus a reduction in the fidelity during reconstruction. Notice that subject 2 has a significant amount of curvature, in comparison to subject 1, with most curvature occurring in the two proximal regions. We surmise that the lower PGD percentage in the distal 1 segment, as compared to that of the proximal 2 segment, is due in part to the more extreme curvature in the proximal segments.

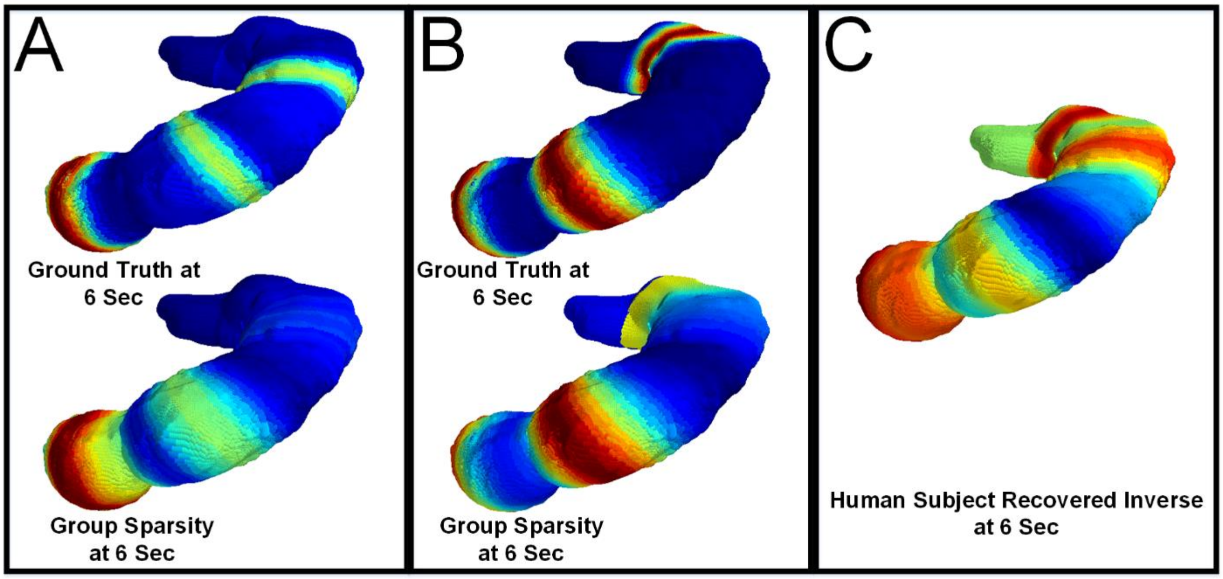

Fig H. Subject 2 3D Model Reconstructed Potentials (A) the second subject stomach ground truth normative simulation and reconstructed potentials at the same time point (6 seconds). (B) the second subject stomach ground truth disordered simulation and reconstructed potentials at the same time point. (C) the second subject reconstructed potentials from the recorded data

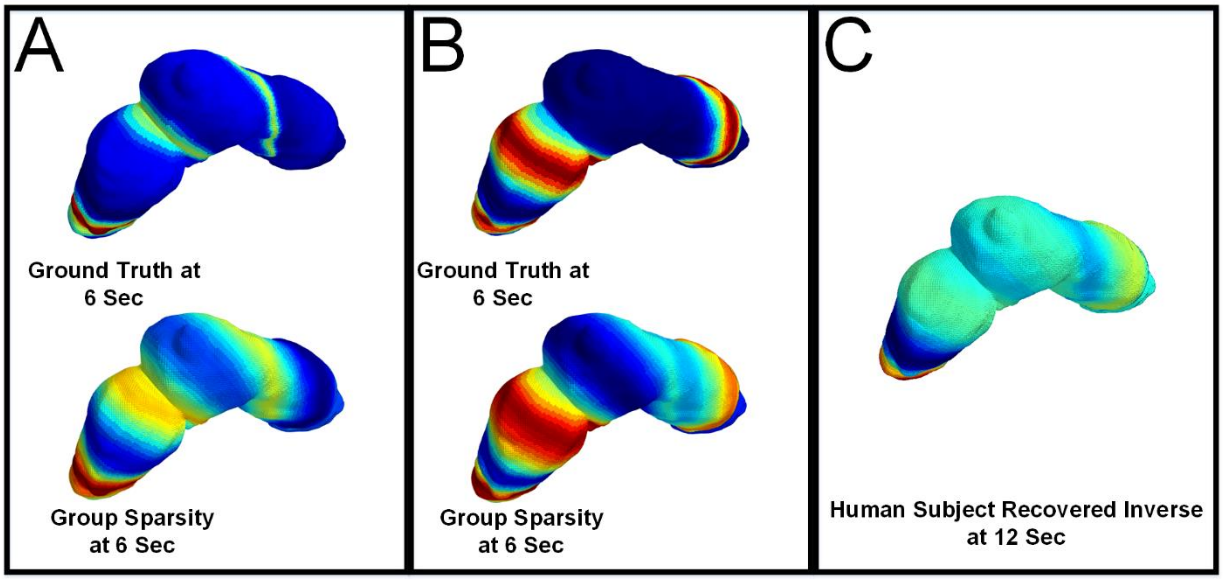

Fig I. Subject 1 3D Model Reconstructed Potentials (A) the first subject stomach ground truth normative simulation and reconstructed potentials at the same time point (6 seconds). (B) the first subject stomach ground truth disordered simulation and reconstructed potentials at the same time point (6 seconds). (C) the second subject reconstructed potentials from the recorded data

Figs H and I show the localization results on the stomach geometry of the two human subjects.

References

1. Gharibans AA, Coleman TP, Mousa H, Kunkel DC. Spatial Patterns From High-Resolution Electrogastrography Correlate With Severity of Symptoms in Patients With Functional Dyspepsia and Gastroparesis. Clinical Gastroenterology and Hepatology. 2019;.
